# Bat rabies in Washington State: Temporal-spatial trends and risk factors for zoonotic transmission (2000–2017)

**DOI:** 10.1371/journal.pone.0205069

**Published:** 2018-10-09

**Authors:** Jesse Bonwitt, Hanna Oltean, Misty Lang, Rochelle M. Kelly, Marcia Goldoft

**Affiliations:** 1 Epidemic Intelligence Service, Division of Scientific Education and Professional Development, Centers for Disease Control and Prevention, Atlanta, Georgia, United States of America; 2 Office of Communicable Disease Epidemiology, Washington State Department of Health, Shoreline, Washington, United States of America; 3 Department of Anthropology, University of Durham, Durham, United Kingdom; 4 Public Health Laboratories, Washington State Department of Health, Shoreline, Washington, United States of America; 5 Department of Biology, University of Washington, Seattle, Washington, United States of America; 6 Burke Museum of Natural History and Culture, University of Washington, Seattle, Washington, United States of America; Wistar Institute, UNITED STATES

## Abstract

**Background:**

Rabies is a zoonotic viral disease that can affect all mammals. In the United States, the majority of human rabies cases are caused by bats, which are the only known reservoirs for rabies virus (RABV) in Washington State. We sought to characterize bat RABV epidemiology in Washington among bats submitted by the public for RABV testing.

**Methods:**

We examined temporal and spatial trends in RABV positivity (% positive) for taxonomically identified bats submitted to diagnostic laboratories during 2006–2017. For a subset of *Myotis* species, we evaluated sensitivity and predictive value positive (PPV) of morphological identification keys, using mitochondrial markers (cytochrome b) as a reference. For bats tested during 2000–2016, we analyzed RABV positivity by circumstances of encounters with humans, cats, and dogs.

**Results:**

During 2006–2017, RABV positivity for all bat species was 6.0% (176/2,928). Among species with ≥100 submissions, RABV positivity was 2.0%–11.7% and highest among big brown bats (*Eptesicus fuscus*). An increasing trend in annual positivity was significant only for big brown bats (*P* = 0.02), and was circumstantially linked to a geographic cluster. Sensitivity and PPV of morphological identification keys was high for *M*. *evotis* but varied for *M*. *lucifugus*, *M*. *californicus*, *M*. *yumanensis*, and *M*. *septentrionalis*. A positive RABV result was significantly associated with nonsynanthropic species, abnormal behavior, abnormal hiding, injury, biting, found in a body of water, found alive, found outdoors, and caught by a dog.

**Conclusion:**

Monitoring passive RABV surveillance trends enables public health authorities to perform more accurate risk assessments. Differences in temporal and spatial trends in RABV positivity by bat species indicate the importance of collecting taxonomic data, although morphological identification can be unreliable for certain *Myotis* species. Current public health practices for RABV exposures should be maintained as RABV infection in bats can never be excluded without diagnostic testing.

## Introduction

Rabies is a zoonotic viral disease caused by viruses of the genus *Lyssavirus*. All mammals are susceptible to infection, and disease is nearly always fatal after clinical onset. Of the more than 16 known *Lyssavirus* species, rabies virus (RABV) is the only one naturally present in the Americas [1, 2]. Bats and wild mesocarnivores constitute major reservoir species [3]. The majority of domestically acquired human rabies cases in the United States are caused by bats. During 2003–2017, 17 of 20 reported primary human rabies cases were associated with bat exposures or bat RABV variants [4, 5].

Transmission of RABV occurs through contact with saliva or neural tissue of a rabid mammal, usually through bites or scratches [6]. Clinical signs include behavioral changes and progressive paralysis, although rarely, neurological signs can be minor or apparently absent. In bats, signs can include diurnal activity, inability to fly, and aggressiveness [6].

Vaccination and postexposure prophylaxis (PEP) are highly effective at reducing mortality in humans, provided that they are administered before symptom onset [7]. In areas with endemic RABV, abnormally acting wild mammals should be considered rabid until proven otherwise [6]. In the event of a human exposure, postmortem diagnostic testing of wild mammals determines the need for PEP. If diagnostic testing is not possible or inconclusive, the requirement for PEP is decided on the basis of a risk assessment. Such integrated exposure management considers rabies epidemiology in the area where the animal-human contact occurred, and type and circumstances of the exposure [8].

While RABV is typically maintained as species-specific variants within a given reservoir, cross-species transmission occurs occasionally, and has been implicated in host shift events leading to the establishment of RABV into new reservoir species [9–13]. Therefore, rabies surveillance in wild mesocarnivores and bats is essential to guide risk assessments, and detect epizootics or changes in RABV reservoir epidemiology that can alter the dynamics of zoonotic transmission.

In the United States, passive RABV surveillance is conducted by public health laboratories that test wild and domestic mammals for diagnostic purposes following a known or likely human exposure to RABV. Of 205,439 bats submitted for RABV testing in the continental United States, 6.7% were positive during 2001–2009 [3], including at least 33 of 41 indigenous bat species [14]. However, the accuracy of species-specific bat rabies surveillance data in the United States can be compromised by inadequate species identification [3, 9, 13]. In particular, *Myotis* species can be difficult to differentiate, due to their similar morphology [13, 15, 16].

Although mesocarnivores such as coyotes (*Canis latrans*), foxes (*Urocyon cineroargenteus*, *Vulpes* spp.), raccoons (*Procyon lotor*), and skunks (*Mephitits mephitis*, *Spilogale putorius*) constitute other RABV reservoirs in North America [1], none are currently known reservoirs in Washington State or bordering jurisdictions (British Columbia, Idaho, and Oregon) [4, 13, 17]. Trends in bat rabies surveillance in North America have been described previously [18–24], but the most recent description of bat rabies epidemiology in the Pacific Northwest dates back to 1985 [25, 26].

The objective of this study was to provide a comprehensive overview of bat RABV in Washington State using passive surveillance data. Specifically, our aims were to (i) compare methods of *Myotis* species identification using morphological keys and genetic identification, (ii) characterize temporal and spatial trends of bat RABV, and (iii) assess risk factors for bat RABV infection by circumstances of encounter.

## Methods

### Bat rabies surveillance

When a known or likely contact with a bat occurs, Washington residents are asked to contact their local health jurisdiction and submit the bat, if available, for RABV testing. This service is provided free of charge at the Washington State Public Health Laboratories (PHL), provided that the exposure meets national criteria for RABV testing [8]. In the absence of human exposure, bats can be tested by Oregon State University at the submitter’s expense. All RABV test results for bats originating from Washington are reportable to the Washington State Department of Health (WADOH). Data concerning bat health and description of the exposure are collected at the time of submission.

### RABV diagnosis and identification of bat species

Postmortem RABV testing was performed by direct fluorescent antibody testing of brain tissue [27]. Results are reported as positive, negative, equivocal (unable to rule-in or rule-out RABV), or unsatisfactory (brain tissue is of unsatisfactory condition to report a valid RABV result).

Bats were identified to species level according to morphological identification keys [28]. Additionally, a convenience sample of 73 *Myotis* spp. submitted for RABV testing were identified genetically using mitochondrial markers by the Burke Museum of Natural History and Culture (S1 Appendix). Briefly, after extraction of DNA from wing tissue samples, we amplified a 654 base pair fragment of the cytochrome b (Cytb) gene by polymerase chain reaction (PCR). Sequences were obtained using an ABI 3730XL sequencer (Applied Biosystems Incorporated, Carlsbad, CA, USA). Consensus sequences were generated with Geneious 9.0.4 (https://www.geneious.com) [29], and aligned using the MUSCLE alignment [30] in MEGA 7 [31]. We used the basic local alignment search tool to compare our *Myotis* spp. sequences to identified reference *Myotis* spp. sequences accessioned in GenBank (https://www.ncbi.nlm.nih.gov/genbank/). Next, we downloaded a total of 24 reference sequences, including 1–2 for each of the *Myotis* species known to occur in Washington State, *M*. *septentrionalis* (not known to occur in WA [25]), and an *Eptesicus fuscus* sequence as an outgroup. We then realigned all our *Myotis* spp. sequences with the reference GenBank sequences using MUSCLE [27] and trimmed all sequences to a common length of 629 base pairs. Finally, we generated a neighbor-joining (NJ) phylogenetic tree of all the CytB sequences in MEGA 7 [28]. Evolutionary distances were calculated using the maximum composite likelihood method [32] and reliability of the nodes were estimated with 1000 non-parametric bootstrap replicates [33].

### Definition of variables

For analysis of temporal and spatial trends, three new categorical variables were created: biogeography was defined relative to the Cascade Range as west (78% of total population, 82 inhabitants/km^2^) or east (22% of total population, 14 inhabitants/km^2^); county size was categorized according to 2010 U.S. Census Bureau data as ≥100,000 inhabitants (12 counties) or <100,000 inhabitants (27 counties); and bats were classified as synanthropic (primarily roost in man-made structures) or nonsynanthropic species (primarily roost in natural structures) according to Klug et al [34] (S1 Table). For circumstances of encounters, submission history was abstracted and coded into predefined categories using standardized definitions (S2 Table) that included bat clinical signs, vital status, location found, and encounters with cats or dogs.

### Statistical analyses

To evaluate bat identification using morphological identification keys, we calculated sensitivity and predictive positive value (PPV) using identification by mitochondrial markers as the reference. For sensitivity, we calculated 95% confidence intervals (95% CI) using exact Clopper-Pearson confidence intervals. For PPV, we calculated 95% CI using logit confidence intervals [35].

For analysis of temporal and spatial trends, we used data from bats submitted for RABV testing during 2006–2017. Bats with incomplete data, equivocal or unsatisfactory RABV test results, or not identified to species level were excluded from analysis. We examined seasonal and annual trends for all bats and for each species with ≥100 submissions. Pearson’s chi-square test or Fisher’s exact test were used to evaluate differences in RABV positivity (percentage test positive) by categorical variables. For all estimates of positivity where N>11, we calculated 95% CI using the modified Wald method. We used Cochrane-Armitage test for trends to test for annual trends in positivity. We applied a negative binomial regression to test for association between positive RABV test result and month, aggregating RABV test positives and total submissions by month and year, and using log of total submissions as offset. For annual and monthly trends, we stratified all analyses by biogeography.

To identify space-time clusters, and assuming that cross-species transmission is uncommon [9], we arbitrarily defined a cluster as ≥4 rabid bats of the same species originating from adjacent counties during contiguous months (e.g., if a rabid bat was found in June, all rabid bats found in that county and adjacent counties during May, June, and July would be considered a cluster).

For circumstances of encounters, we used available data from bats submitted for RABV testing at PHL during 2000–2016. Bats with incomplete data, or equivocal or unsatisfactory results, were excluded from analysis. We used logistic regression to calculate unadjusted odds ratios (ORs) for RABV positivity by circumstances of encounters. For bats found alive, we calculated adjusted ORs for RABV positivity by clinical signs. For all statistical analyses, an alpha of p <0.05 was considered significant. Analyses were performed using SAS 9.4 (SAS Institute Incorporated, Cary, NC, USA) and Microsoft Excel 2013 (Microsoft Corporation, Redmond, WA, USA). This study was reviewed by CDC for human subjects protection and was deemed to be nonresearch.

## Results

### Species identification

Sensitivity of morphological identification was high for California myotis (100%, 95% CI: 48–100), western long-eared myotis (90%, 95% CI: 73–98), and Yuma myotis (75%, 95% CI: 35–97). The PPV of morphological identification was high for western long-eared myotis (100%), and little brown bats (94%, 95% CI: 70–99), but was low for Yuma myotis (32%, 95% CI: 20–46). Both sensitivity and PPV of morphological identification was low for northern long-eared myotis (0%) (Table 1 and S4 Fig). Morphologically identified Yuma myotis were identified as little brown bats using mitochondrial markers. Morphologically identified California myotis were identified as Yuma myotis and little brown bats using mitochondrial markers.

**Table 1:** Number of *Myotis* spp. bats identified with morphological keys (rows) and mitochondrial markers (columns). Sensitivity and predictive value positive of morphological identification are calculated with mitochondrial markers as the reference.

Identification by morphological keys	Identification by mitochondrial markers					Sensitivity		Predictive value positive	
	*M*. *californicus* *M*. *ciliolabrum*	*M*. *evotisM*. *keeniM*. *thysanodes*	*M*. *lucifugus*	*M*. *yumanensis*	Total	%	95% CI	%	95% CI
California myotis(*M*. *californicus*)	5		0	2	7	100	48–100	71	39–91
Western long-eared myotis(*M*. *evotis*)		26			26	90	73–98	100	
Little brown bat(*M*. *lucifugus*)		1	17		18	55	36–73	94	70–99
Yuma myotis(*M*. *yumanensis*)			13	6	19	75	35–97	32	20–46
Northern long-eared myotis(*M*. *septentrionalis*)		2			2	0	0	0	
Keen’s Myotis(*M*. *keeni*)		1			1				
Total	5	29	30	8	73				

### Temporal and spatial trends in bat rabies

#### Number of observations

A total of 3,481 bats were submitted for RABV testing during 2006–2017, of which 180 (5.2%) were positive. In total, 553 bats (15.9%), including 4 rabid bats, were excluded from the analysis, and 2,928 were included. Reasons for exclusion included incomplete submission data (10 bats [0.3%]); equivocal or unsatisfactory RABV test result (259 bats [7.4%]); not identified to species level (201 bats [5.8%]); and both equivocal or unsatisfactory test result and not identified to species level (83 bats [2.4%]). Of the 3,481 bats submitted for RABV testing, 3,187 (91.5%) bats were identified to species level, but the remainder were too physically damaged or immature to perform morphological identification.

#### Species tested and RABV positivity

RABV positivity was 6.0% (176 bats, 95% CI: 5.2–6.9). Ten of 16 species tested had ≥1 rabid bat (Table 2). Of all bat species tested, the three species with highest RABV positivity were all nonsynanthropic species with the lowest numbers of submissions: hoary bats (4/15, 27%, 95% CI: 10–52), northern long-eared myotis (4/23, 17%, 95% CI: 6–38), and Townsend’s big-eared bats (1/6, 17%). Among the 6 bat species with ≥100 submissions, the highest positivity was in big brown bats (11.7%, 95% CI: 9.7–14.1), silver-haired bats (8.8%, 95% CI: 5.8–13.1), and western long-eared myotis (7.3%, 95% CI: 4.3–11.9%). Positivity among all nonsynanthropic species was 9.1% (44/482, 95% CI: 6.8–12.0) and was significantly higher (*P* = 0.002) compared with synanthropic species (132/2,446, 5.4%, 95% CI: 4.6–6.4).

**Table 2:** Number of identified bats with definitive RABV test result (N = 2,928), number of rabid bats, and bat RABV positivity by species identified by morphological keys, Washington State—2006–2017.

Species	No. bats tested	No. bats positive	% bats positive (95% CI)	Surveillance % bats positive (number bats tested)		
				Passive		Active
				United States 2001–2009†	British Columbia 1971–1985‡	North America 1955–2011^±^
**Species with ≥100 submissions**						
Big brown bat (*Eptesicus fuscus)*	819	96	11.7 (9.7–14.1)	4.7 (65,167)	16.8 (197)	2.3 (1,146)
Silver-haired bat (*Lasionycteris noctivagans*)	239	21	8.8 (5.8–13.1)	8.3 (1,367)	14.6 (41)	1.0 (105)
California myotis (*Myotis californicus*)	788	16	2.0 (1.2–3.3)	4.5 (552)	6.2 (145)	0 (21)
Western long-eared myotis (*Myotis evotis*)	193	14	7.3 (4.3–11.9)	6.1 (295)	10.2 (59)	0 (28)
Yuma myotis (*Myotis yumanensis*)	403	10	2.5 (1.3–4.6)	8.1 (148)	3.0 (33)	0 (61)
Little brown bat (*Myotis lucifugus*)	380	8	2.1 (1.0–4.2)	2.2 (10,877)	2.7 (300)	0.1 (2,235)
**Species with** <**100 submissions**						
Hoary bat (*Lasiurus cinereus*)	15	4	27 (10–52)	35.3% (598)	20.0% (10)	1.1% (182)
Northern long-eared myotis (*Myotis septentrionalis*)^¶^	23	4	17 (6–38)	4.5% (202)	0	0
Fringed myotis (*Myotis thysanodes*)	35	2	6 (0–20)	12.5% (8)	0	0% (21)
Townsend’s big-eared bat (*Corynorhinus townsendii*)	6	1	17	21.9% (32)	0% (4)	3.9% (51)
Spotted bat (*Euderma maculatum*)	1	0	0	50% (2)	0	0
Southern red bat (*Lasiurus blossevillii*)^¶^	1	0	0	0% (34)	0	NA
Small-footed myotis (*Myotis ciliolabrum*)	13	0	0 (0–27)	0% (4)	0	NA
Keen’s myotis (*Myotis keenii*)	3	0	0	3.0% (267)	0% (6)	0
Long-legged myotis (*Myotis volans*)	8	0	0	30% (10)	0% (2)	0% (23)
Big free-tailed bat (*Nyctinomops macrotis*)^¶^	1	0	0	42.9% (21)	0	0% (31)
Pallid bat (*Antrozous pallidus*)	0	0	0	8.9% (452)	0% (2)	0% (52)
Canyon bat (*Parastrellus hesperus*)	0	0	0	21.7% (750)	0	0% (70)
**All species**	**2,928**	**176**	**6.0 (5.2–6.9)**	**4.8% (80,786)**	**8.1% (799)**	**1.3% (4,026)**

^¶^Not known to be indigenous to Washington.

Data from Klug et al (2011)^±^, Patyk et al (2011)^†^, and Prins et al (1988)^‡^.

#### Seasonal trends

**Fig 1:**
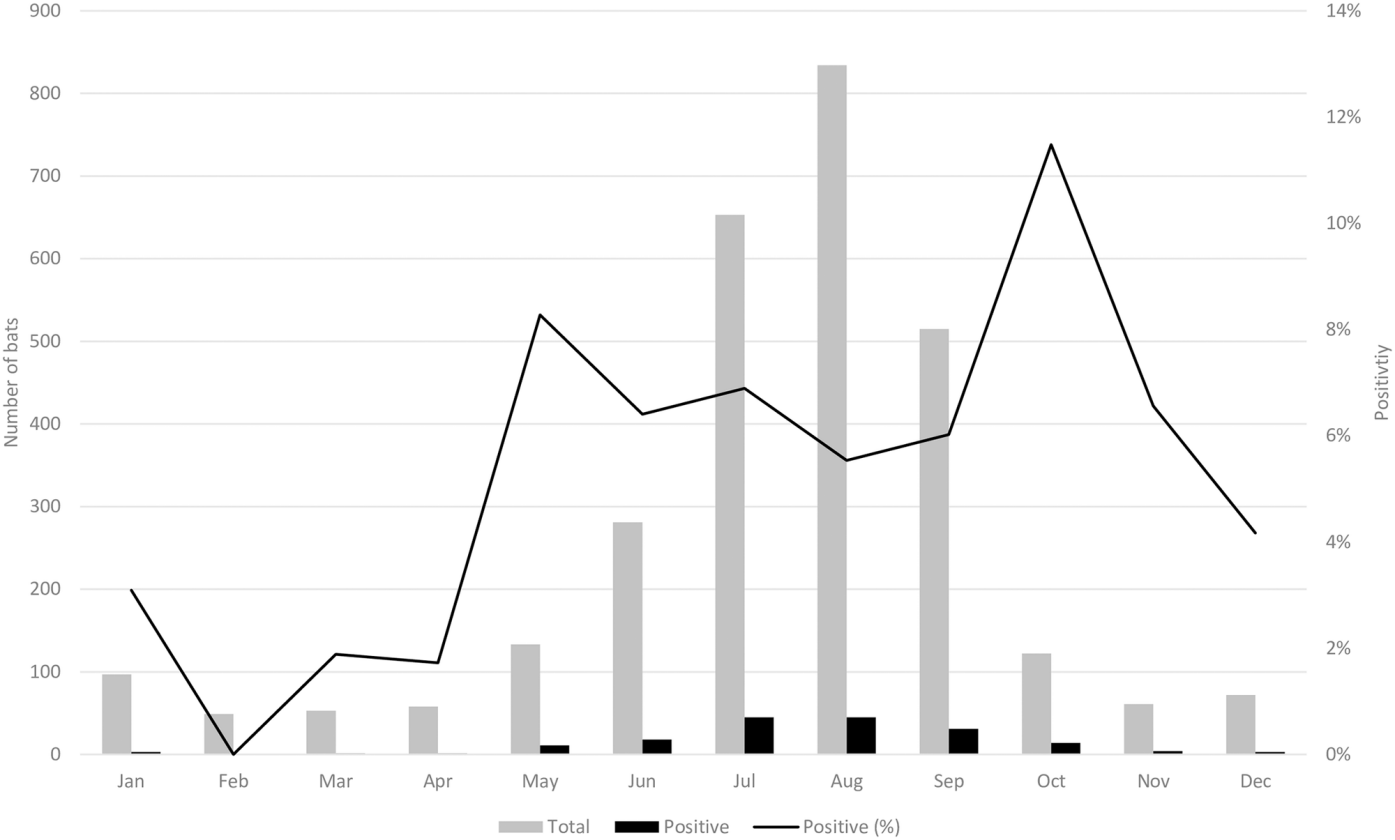
Numbers of identified bats with definitive RABV test results (N = 2,928) and RABV positivity by month, Washington State—2006–2017.

**Fig 2:**
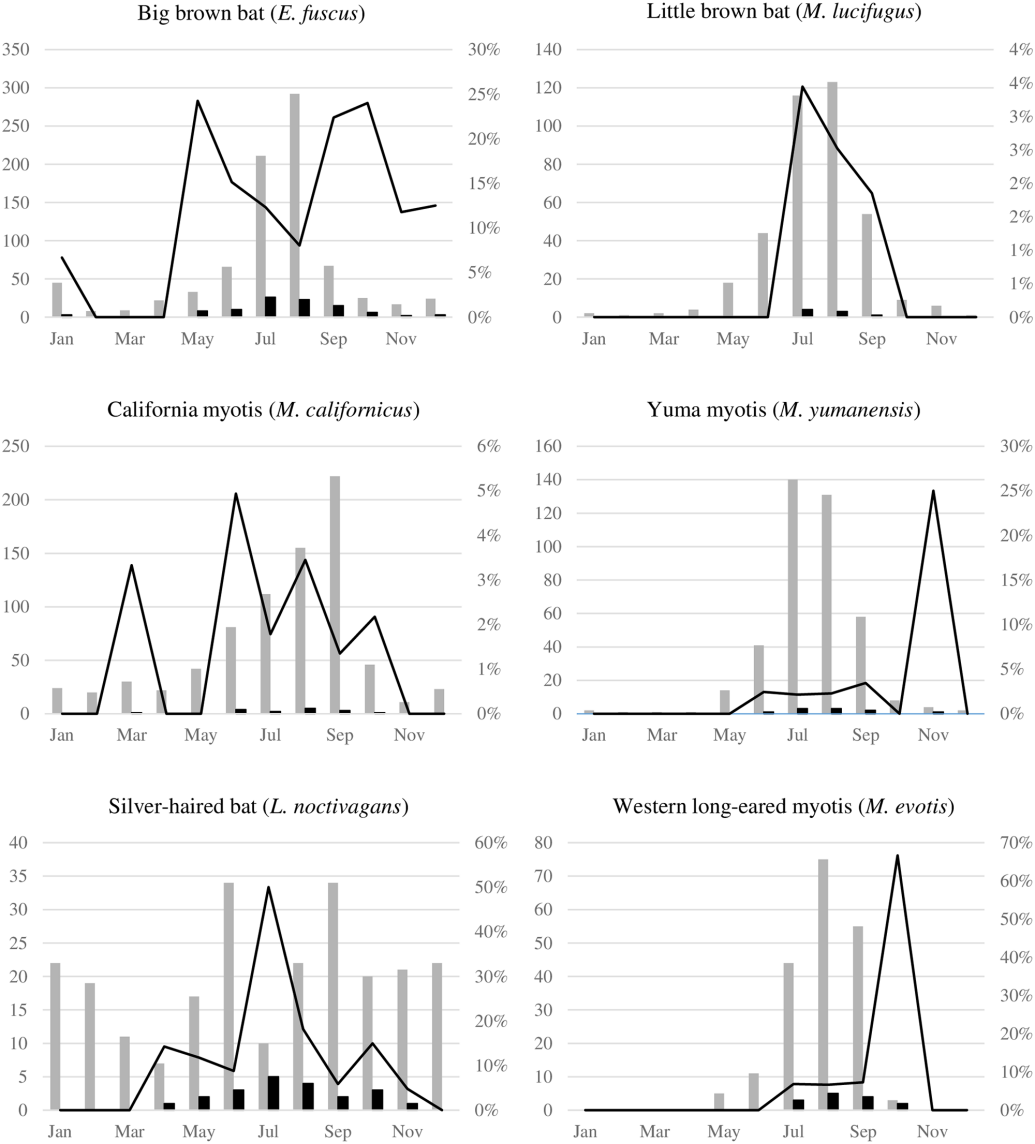
Numbers of bats tested (grey bars), number of RABV positive bats (black bars), and RABV positivity (black line) by month for bat species with ≥100 submissions and definitive test result, Washington State—2006–2017.

For all identified bats submitted for RABV testing, 2,538 (87%) were submitted during May–October. Among the 176 rabid bats, 164 ((93.2%, 95% CI: 88.3–96.2) tested positive during this same period. Positivity by month for all identified species was bimodal (peak in May and October), ranging from 0% (0/49 bats, 95% CI: 0–8.7) in February to 11.5% (14/122 bats, 95% CI: 6.8–18.5) in October (Fig 1 and S1 Fig). However, the count of rabid bats by month was unimodal, peaking during July and August. When stratified by species, positivity by month was unimodal, bimodal, or multimodal, and peaked at different months (Fig 2). For certain species, the monthly count of rabid bats differed from that of the monthly positivity.

Differences in monthly positivity in little brown bats, California myotis, and silver-haired bats were not statistically significant. Insufficient data were available for other species, or when stratified by biogeography. However, time series plots of bat submissions and rabid bats displayed a convincing cyclical pattern, in particular for little brown bats, western long-eared myotis, California myotis, and Yuma myotis (S2A–S2F Fig).

#### Annual trends

**Fig 3:**
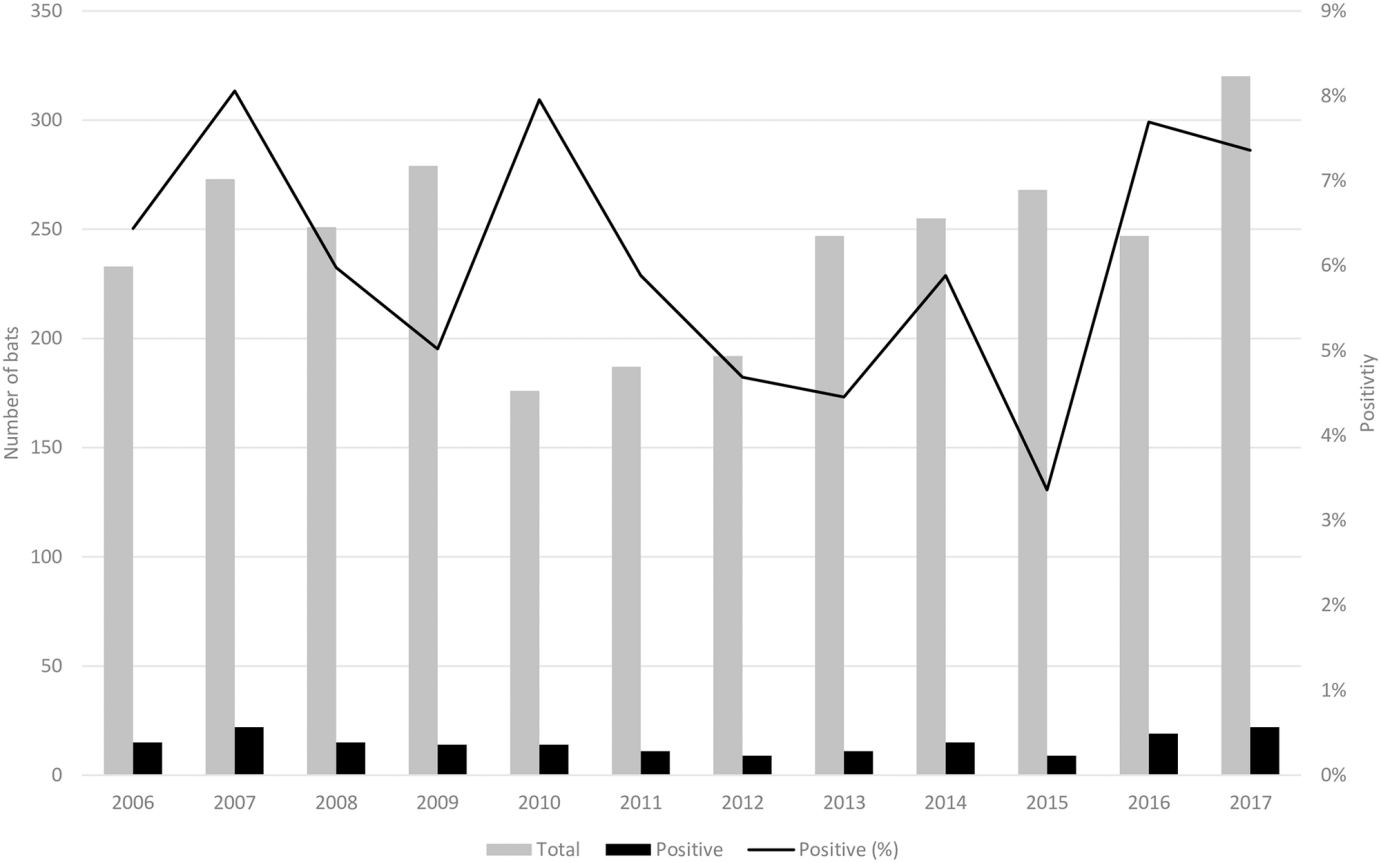
Numbers of identified bats with definitive RABV test results (N = 2,928) and RABV positivity by year, Washington State—2006–2017.

Annual RABV positivity for all species ranged from 3.4% (9/268 bats, 95% CI: 1.7–6.3) to 8.1% (22/273 bats, 95% CI: 5.3–11.9) (Fig 3 and S1 Fig) and a significant trend was not detected from 2006 to 2017 (*P* = 0.67), including when stratified by biogeography. When stratified by species with ≥100 submissions, annual RABV positivity varied. A significant trend was only observed for big brown bats (*P* = 0.03). Positivity increased from an average of 10.1% (69/681 big brown bats, 95% CI: 8.0–12.7)) during 2006–2015 to 20.5% (27/138 big brown bats, 95% CI: 13.8–27.0) during 2016–2017.

#### Spatial trends

In total, 38 of 39 Washington counties submitted bats for testing during 2006–2017. The median number of bats submitted by county per year was 3 (range: 0–54). The majority of bats were submitted from west of the Cascade Range (81.6%; 2,389 bats), including when stratified by species. No significant difference was reported in RABV positivity west (5.7%) and east (7.2%) of the Cascade Range (*P* = 0.19), but a significant interaction between biogeography and county population size (*P* = 0.02) was identified. When stratified by population size, RABV positivity in counties with <100,000 inhabitants was significantly higher east of the Cascade Range, compared with counties west of the Cascade Range (OR: 1.7, 95% CI: 1.1–3.2; *P* = 0.03). For counties ≥100,000 inhabitants, positivity did not differ by biogeography (OR: 0.7; 95% CI: 0.4–1.3; *P* = 0.27). Stratification by species and biogeography resulted in too few observations for analysis.

#### Space-time clustering

We identified one defined cluster involving long-eared myotis (4 bats), and five clusters involving big brown bats (4–11 bats) (S3 Table). Geographic coordinates could be retrieved for rabid bats in the 2017 cluster (11 big brown bats), of which 10 were located within a radius of ~40 km (S3 Fig).

### Circumstances of bat encounters

#### Number of observations

Submission histories regarding the circumstances of bat encounters were available for 3,466 bats received for RABV testing during 2000–2016, of which 210 (6.1%) were positive. Bats excluded from the analysis had equivocal or unsatisfactory test results (343 bats [9.9%]), or circumstances of encounter were either unknown or could not be assigned to a specific category (314 bats [9.1%]). The final number of bats included in the analysis was 2,809, of which 190 (6.8%) had RABV-positive test results.

#### Positivity and odds ratio by circumstance of bat encounters

Injury (336 bats) and biting (146 bats) were the most frequently reported signs (Table 3). The highest RABV positivity was among bats exhibiting biting (28.8%), abnormal behavior (21.5%), and found in a body of water (19.0%). Among live bats, all clinical signs were significantly associated with RABV positivity (Table 3). In the multivariate analysis, point estimates for clinical signs differed only slightly from that of the univariate analysis, and all variables remained significant. Of 2,809 bats with known vital status, the majority were found alive (2,516 bats [89.6%]) and had higher odds of testing positive for RABV, compared with those found dead (OR: 3.3; 95% CI: 1.5–7.6). A location by circumstance of encounter was available for 2,124 bats. The most common location was in a house (86.1%; 1,828 bats). However, bats found outdoors had 4 times higher odds of being rabid, compared with those found inside a house (OR: 4.0; 95% CI: 2.5–6.4). Bats captured by cats or dogs accounted for 369 submissions. Although cats caught a higher percentage of bats (330 bats [89.4%]), dogs had higher odds of catching rabid bats (OR: 3.8; 95% CI: 1.4–10.5).

**Table 3:** Number of bats with definitive test result (N = 2,809), number and percent of bats positive for RABV, and odds ratio for RABV positivity, Washington State—2000–2016. Categories of bat signs and behaviors are not mutually exclusive. Bold font indicates statistical significance.

	No. bats tested	No. bats rabies positive	% bats rabies positive	Odds ratio	(95% CI)	*P* value
**Bat Clinical Signs**						
Alive, no abnormal behavior	2,427	158	6.5	Reference		
Abnormal behavior	121	26	21.5	**3.9**	**2.5–6.2**	**<0.0001**
Alive, no abnormal hiding	2,512	178	7.1	Reference		
Abnormal hiding	36	6	16.7	**2.6**	**1.1–6.4**	**0.03**
Alive, not injured	2,182	127	5.8	Reference		
Injured	366	57	15.6	**3.0**	**2.1–4.2**	**<0.0001**
Alive, no bite	2,402	142	5.9	Reference		
Bite	146	42	28.8	**6.4**	**4.3–9.5**	**<0.0001**
Not in a body of water	2,788	186	6.7	Reference		
In a body of water	21	4	19.0	**3.3**	**1.1–9.9**	**0.03**
**Vital Status**						
Dead	261	6	2.3	Reference		
Alive (downed)	2,516	183	7.3	**3.3**	**1.5–7.6**	**<0.05**
Alive (inflight collision)	32	1	3.1	1.4	0.2–11.8	0.8
**Location of Bat Encounter**						
Inside house	1,828	76	4.2	Reference		
Inside other structure	101	6	5.9	1.5	0.6–3.4	0.4
Outdoors	195	29	14.9	**4.0**	**2.5–6.4**	**< 0.05**
**Bat Interaction with Household Pets**						
Caught by cat	330	15	4.5	Reference		
Caught by dog	39	6	15.4	**3.8**	**1.4–10.5**	**< 0.05**

## Discussion

### Temporal and spatial trends in bat rabies

In Washington, positivity for all species (6.0%) was lower than that reported from passive surveillance in British Columbia during 1971–1985 (8.6%) [25], Idaho during 1999–2016 (10.6%) [36], and Oregon during 2006–2015 (7.6%) [37]. RABV positivity in bats tested at public health agencies throughout the United States during 2001–2009 was 6.7% (13,693 rabid bats), although this decreased to 4.8% when adjusted to include only species indigenous to Washington [3] (Table 2).

Bats with the highest positivity (≥15%) were all nonsynanthropic species, however these were submitted in limited numbers (≤23 bats per species), which might affect the accuracy of species-specific positivity rates. When grouped by synanthropic status, nonsynanthropic species also had significantly higher odds of being rabid than synanthropic ones. Because they tend to avoid human activity, nonsynanthropic bats might be more likely to come into contact with humans and be submitted for testing when rabid. The subsequent bias might thus lead to considerable overestimation of RABV prevalence in passively surveilled nonsynanthropic species [34], including in this study.

When all species were combined, positivity and number rabid tended to be highest during May–October, with a peak in July–August (number rabid) and October (positivity), a finding broadly in agreement with findings in North America [3, 18–21, 23, 38] and in European bat lyssaviruses [39]. This is consistent with the observation that most cases of bat-associated human rabies occur during September–December, anticipating a 1–2 months incubation period in humans [40]. However, no statistically significant seasonal trend in positivity was discernable, which might be caused by the extremely low denominator during November–April. The reduced number of bat submissions during this period likely reflects both bat and human behaviors, because most bat species either undergo hibernation, torpor [41], or migrate out-of-state during October–April [41], concurrent with a decrease in outdoors human activity during this period [19, 38]. In this regard, submission of silver-haired bats and California myotis provides an insight as to whether the observed decrease in RABV positivity during November–April can be attributed to a seasonal sampling bias or a true seasonality in RABV prevalence. Both species were submitted throughout the year, consistent with their tolerance for colder temperatures and intermittent emergence from torpor or hibernation to hunt [28, 42]. However, no rabid bats were documented during December–March (silver-haired bats) or November–February (California myotis), indicating that seasonal RABV positivity cannot be solely attributed to seasonal submission bias in these species.

The differences in RABV epidemiology among bat species probably reflect their diverse natural history. In particular, factors affecting contact rates, such as hibernation, torpor, seasonal dispersion, reproduction, and social structure might determine species-specific RABV dynamics [18–20, 39, 43, 44], although variability in pathogenicity and transmissibility of RABV variants, and disease susceptibility and immunity among hosts [40, 45], might also be at play.

The only significant annual trend was among big brown bats, in which positivity approximately doubled during 2016–2017, compared with 2006–2015. Schowalter [44] hypothesized that RABV among gregarious sedentary bats, such as big brown bats, could be maintained through epizootics and would more likely be detected through passive surveillance. Our circumstantial finding of geographic clustering during this same period might possibly be indicative of a local RABV epizootic in big brown bats starting during 2016.

Big brown bats stand out as a major RABV reservoir in Washington and the only synanthropic species with a far higher positivity (11.7%) than other synanthropic species (2.2%). Big brown bats are broadly distributed throughout Canada and the United States [46], allowing for comparison throughout the continent. In a review of RABV positivity in North America during 1955–2011, positivity among actively sampled big brown bats was 2.3% (26/1,146). Among passively submitted big brown bats, positivity ranged between 4.7% (3,040/65,167) [3] and 5.9% (1,695/28,905) [34]. Among the literature reviewed by Klug et al [34], and taking into account recent publications [20], a positivity ≥7% among big brown bats has only previously been reported from passive surveillance in southern Canada during 1963–1985 (S4 Table).

The observed seasonality in RABV prevalence among big brown bats is consistent with ecological modeling [43] and field studies [47] that suggest that seasonal variations in RABV prevalence can be attributed to seasonal aggregation and birthing in maternal colonies, the subsequent introduction of immunologically naïve individuals into colonies, and their dispersal upon maturity.

Reasons for high RABV positivity among big brown bats in Washington are unknown. Big brown bats can form large maternity colonies up to several hundred individuals [41, 48], which might facilitate intraspecific RABV transmission. However, this alone is an insufficient explanation, as little brown bats are also highly colonial [41], but RABV positivity is thought to be low in this species, consistent with our findings [13]. Alternatively, big brown bats might be more likely to be submitted for testing by virtue of their synanthropic disposition. However, in this case a high positivity among other synanthropic species would also be expected. The high positivity among big brown bats is further surprising because the RABV variant associated with big brown bats has only been identified once in humans [5, 40], notably in one of two people known to have died from rabies in Washington since the 1950s [49, 50].

### Circumstances of bat encounters

Abnormal behavior and hiding, injury, biting, and found in a body of water were all associated with RABV positivity. These results are consistent with the pathogenesis of RABV in mammals [6] and findings from previous studies, which include aggression, ataxia, disorientation, or lethargy [24]; downed, or described as ill or aggressive [38]; and biting [19, 22]. Although biting was not associated with RABV positivity in one study [38], 17%–39% of human rabies cases with bat RABV variant in Canada and the United States were bitten by bats [51, 52]. However, these signs cannot be considered exclusively as indicators of RABV infection, because healthy bats display biting, paralysis, and tremors when threatened or attacked [14]. Drowning has only been associated circumstantially with rabies [44] and might be caused by neurological impairment affecting flight when bats skim water surfaces during foraging and drinking.

Rabid bats were more likely to be found alive and flightless than found dead, and more likely to be found outdoors than inside a house, consistent with previous reports [21, 38]. Both these findings likely reflect public health testing criteria, because contact with live bats and those found indoors (e.g., sleep exposures) are considered exposures that warrant testing according to national guidelines [8]. Bats submitted for RABV testing were disproportionally found indoors (86.1%), a finding that also likely reflects a submission bias, as healthy bats trapped indoors are presumably easier to catch than healthy free-flying bats outdoors.

Although more cats were in contact with bats, dogs had a nearly 4 times higher odds of being exposed to rabid bats than cats, consistent with previous reports [23]. This is possibly because cats are more efficient predators of healthy flying bats compared to dogs, whereas the latter presumably might catch mostly downed bats. These findings underscore the importance of maintaining up-to-date RABV vaccination throughout North America where rabies is endemic.

### Limitations

The ability to identify bats using morphological keys is uneven between species and a low identification rate can bias results towards species that are easiest to identify. This bias was limited in our study as 91.5% of bats were identified to species level, compared with 42.6% of bats submitted for RABV testing across the United States [3]. However, a large proportion of California myotis, Yuma myotis, and little brown bats were misidentified. The northern long-eared myotis is also likely to have been misidentified, as its geographic range is not known to extend into Washington [53], and positivity (17.4%) was nearly 4 times that of the national average (4.5%) [3]. While misidentification of certain *Myotis* species is expected [15, 16], our findings further indicate that bat RABV surveillance data should be interpreted with caution if morphological keys are used for these *Myotis* species [13]. Misidentification of bats included in our study might explain why our results differ from the conclusions of a phylogenetic study, which found that California myotis, Keen’s myotis, Western long-eared myotis, and Yuma myotis were the principal RABV reservoirs among the *Myotis* species inhabiting British Colombia [13]. In contrast, among *Myotis* species with ≥100 submissions, RABV positivity for California myotis and Yuma myotis was lower than the national average (Table 2).

Multiple factors can bias submission of bats for RABV testing, including population density [20, 21] and public awareness [20, 21], especially in urban settings. Both these factors might have affected our results, as bat submissions were predominantly from west of the Cascade Range (78% of total population in Washington), an area where people may have exhibited increased reporting, leading to observed space or time clustering. Underreporting of bat exposures might also have affected our results: in Canada, fewer than 5% (N = 41) of persons with potential sleeping exposure to bats sought medical attention [54], and in Illinois, only 25%–54% of visits to emergency departments related to rabies were reported to a county health department [55].

Prevalence of RABV in healthy bat populations is likely to be overestimated using passive surveillance, as only animals with suspected RABV infection that have exposed people or domestic animals are tested. In contrast, random sampling (active surveillance) indicates that RABV prevalence in North American bats is closer to 0.8%, or 1.3% when including only species indigenous to Washington [34] (Table 2). However, active surveillance is resource-intensive and might underestimate RABV prevalence if clinically ill bats are missed during sampling due to hiding or other abnormal behaviors [18, 34]. Diagnostic testing also provides a practical and cost-efficient means to passively monitor trends in bat RABV in a timely manner, as long as results are interpreted with consideration of sampling bias.

The small number of samples might have affected our results and led to our failure to demonstrate statistical significance, especially as we decided to stratify by species. Space-time clusters were difficult to demonstrate because we lacked geographical coordinates. Because we used county as the highest resolution, spatial clusters in counties with a large geographic area might not represent true clusters as RABV positive bats might be randomly dispersed throughout the area. Significant trends in seasonality were difficult to demonstrate because of limited sample sizes when stratifying by species and county, months, or years. However, we maintain that examining RABV trends by species is important given that the diversity in their life histories may influence on RABV dynamics among species. Unfortunately, limited data are available on bat ecology in Washington, precluding us from further contextualizing our findings with respect to the unique life history, population density, and distribution of each species. Finally, characterizing RABV variants could have better contextualized our findings through use of molecular epidemiology.

## Conclusions

Analysis of passive bat RABV surveillance in Washington indicates that big brown bats are a major reservoir, and circumstantial evidence suggests the possible presence of an epizootic during 2016–2017 west of the Cascade Range. Whereas certain species, ecological characteristics (nonsynanthropic species), seasons, and circumstances of encounters present a heightened risk of bat RABV infection, none of these variables can conclusively exclude bat RABV infection. Contact with bats should therefore always warrant a public health evaluation and current public health practices for RABV exposures in humans, cats, and dogs, should be maintained.

The epidemiology of bat RABV is not static, and is likely to evolve in accordance with host ecology. In the Pacific Northwest, two recent developments underscore the importance of monitoring RABV trends, which can ultimately alter the risk of zoonotic transmission. First, white-nose syndrome, an emerging fungal pathogen of bats associated with extensive population decline in several bat species, was first reported in Washington during 2016 [56], with unknown consequences on bat RABV dynamics [4]. Second, the Brazilian free-tailed bat (*Tadarida brasiliensis*), which is not indigenous to Washington but accounts for the highest RABV positivity in the United States [3], was reported for the first time in British Columbia during 2015 [57]. These developments further underscore the importance of collecting taxonomic data as part of RABV surveillance to detect changes in bat RABV ecology, such as the introduction of novel bat RABV variants into established populations. As new assays for RABV diagnostic testing become available [58], concurrent identification by genetic barcoding might become increasingly feasible, and provide further insights into the eco-epidemiology of bat-associated RABV.

## Supporting information

S1 Appendix Detailed methods for genetic identification of bats. (PDF)

S1 Table Classification of bats according to synanthropic status. Data from Klug et al (2011)^±^ and Hayes et al (2013)^§^.
(PDF)

S2 Table Definitions for circumstances of encounters between bats and humans, cats, and dogs. (PDF)

S3 Table Bats with positive RABV test results by cluster, Washington State—2006–2017. A space-time cluster was defined as ≥4 RABV positive bats originating from proximal counties during contiguous months.
(PDF)

S4 Table RABV positivity (by fluorescent antibody testing) among passively sampled big brown bats in Southern Canada, 1963–1985. (PDF)

S1 Fig Numbers of identified bats tested with definitive RABV test results (upper figure) and number of identified bats positive for RABV (lower figure) by month and year, Washington State—2006–2017. (PDF)

S2 Fig Numbers of identified bats tested with definitive RABV test results (upper figure) and number of bats positive for RABV (lower figure) by month and year, and by species with ≥100 submissions, Washington State—2006–2017. (PDF)

S3 Fig Cluster of RABV positive big brown bats (*Eptesicus fuscus*) in 2017 (N = 11), Western Washington. Shapes denote month of submission; triangle: May; diamond: June; square: July, circle: August.
(PDF)

S4 Fig Myotis spp. (n = 73) neighbor-joining phylogenetic tree. A neighbor-joining tree generated for 97 CytB sequences of 629bp in length. The 73 *Myotis* spp. sequences generated in this study were morphologically identified by the Department of Health and are indicated by their sample IDs, whereas the 24 reference sequences are indicated by their GenBank accession numbers and species names. Species assigned to each major clade are indicated, and bootstrap values greater than 70% are indicated. Individuals that clustered in clades outside of their morphological assignment are indicated by (*).
(PDF)

## References

[1] Velasco-Villa A, Mauldin MR, Shi M, Escobar LE, Gallardo-Romero NF, Damon I, et al The history of rabies in the Western Hemisphere. Antiviral research. 2017;146:221–32. 10.1016/j.antiviral.2017.03.013 28365457 PMC5620125

[2] ICTV. Rhabdoviridae Genus: Lyssavirus. ICTV Virus Taxonomy. 2018 [cited Feburary 2018. https://talk.ictvonline.org/ictv-reports/ictv_online_report/negative-sense-rna-viruses/mononegavirales/w/rhabdoviridae/795/genus-lyssavirus.

[3] Patyk K, Turmelle A, Blanton JD, Rupprecht CE. Trends in national surveillance data for bat rabies in the United States: 2001–2009. Vector borne and zoonotic diseases. 2012;12(8):666–73. 10.1089/vbz.2011.0839 22607069

[4] Birhane MG, Cleaton JM, Monroe BP, Wadhwa A, Orciari LA, Yager P, et al Rabies surveillance in the United States during 2015. J Am Vet Med Assoc. 2017;250(10):1117–30. 10.2460/javma.250.10.1117 28467751

[5] CDC. Human Rabies 2018 [https://www.cdc.gov/rabies/location/usa/surveillance/human_rabies.html.

[6] Rupprecht CE. Overview of Rabies: Merck Veterinary Manual; 2018 [January 2018]. http://www.merckvetmanual.com/nervous-system/rabies/overview-of-rabies.

[7] Franka R. Rabies In: Heymann D, editor. Control of Communicable Diseases Manual. 18th ed Washington DC, USA: American Public Health Association; 2004.

[8] Manning SE, Rupprecht CE, Fishbein D, CA H, Lumlertdacha B, Guerra M, et al Human rabies prevention—United States, 2008: recommendations of the Advisory Committee on Immunization Practices. MMWR Recomm Rep. 2008;23(57(RR-3)):1–28. 18496505

[9] Streicker DG, Turmelle AS, Vonhof MJ, Kuzmin IV, McCracken GF, Rupprecht CE. Host Phylogeny Constrains Cross-Species Emergence and Establishment of Rabies Virus in Bats. Science. 2010;329(5992):676 10.1126/science.1188836 20689015

[10] Wallace RM, Gilbert A, Slate D, Chipman R, Singh A, Cassie W, et al Right Place, Wrong Species: A 20-Year Review of Rabies Virus Cross Species Transmission among Terrestrial Mammals in the United States. PloS one. 2014;9(10):e107539 10.1371/journal.pone.0107539 25295750 PMC4189788

[11] Leslie MJ, Messenger S, Rohde RE, Smith J, Cheshier R, Hanlon C, et al Bat-associated Rabies Virus in Skunks. Emerging infectious diseases. 2006;12(8):1274–7. 10.3201/eid1208.051526 16965714 PMC3291214

[12] Kuzmin IV, Shi M, Orciari LA, Yager PA, Velasco-Villa A, Kuzmina NA, et al Molecular Inferences Suggest Multiple Host Shifts of Rabies Viruses from Bats to Mesocarnivores in Arizona during 2001–2009. PLoS pathogens. 2012;8(6):e1002786 10.1371/journal.ppat.1002786 22737076 PMC3380930

[13] Nadin-Davis S, Alnabelseya N, Knowles MK. The phylogeography of Myotis bat-associated rabies viruses across Canada. PLoS neglected tropical diseases. 2017;11(5):e0005541 10.1371/journal.pntd.0005541 28542160 PMC5453604

[14] Constantine DG. Bat rabies and other Lyssavirus infections. Reston, Va: U.S. Geological Survey 2009. Contract No.: Circular 1329.

[15] Luszcz TMJ, Rip JMK, Patriquin KJ, Hollis LM, Wilson JM, Clarke HDM, et al A Blind-Test Comparison of the Reliability of Using External Morphology and Echolocation-Call Structure to Differentiate Between the Little Brown Bat (*Myotis lucifugu*s) and Yuma Myotis (*Myotis yumanensis*). Northwestern Naturalist. 2016;97(1):13–23.

[16] Rodhouse TJ, Scott SA, Ormsbee PC, Zinck JM. Field identification of *Myotis yumanensis* and *Myotis lucifigus*: a morphological evaluation. Western North American Naturalist. 2008;68(4):437–43.

[17] BCCDC. British Columbia Annual Summary of Reportable Diseases 2016. British Columbia Centre for Disease Control; 2017.

[18] Burnett CD. Bat rabies in Illinois: 1965 to 1986. 1989(0090–3558 (Print)). 10.7589/0090-3558-25.1.10 2915390

[19] Childs JE, Trimarchi CV, Krebs JW. The epidemiology of bat rabies in New York State, 1988–92. Epidemiology and infection. 1994;113(3):501–11. 7995360 10.1017/s0950268800068515 PMC2271321

[20] Gilbert AT, McCracken GF, Sheeler LL, Muller LI, O’Rourke D, Kelch WJ, et al Rabies Surveillance Among Bats in Tennessee, USA, 1996–2010. J Wildl Dis. 2015;51(4):821–32. 10.7589/2014-12-277 26251992

[21] Mayes BC, Wilson PJ, Oertli EH, Hunt PR, Rohde RE. Epidemiology of rabies in bats in Texas (2001–2010). J Am Vet Med Assoc. 2013;243(8):1129–37. 10.2460/javma.243.8.1129 24094260

[22] Pape WJ, Fitzsimmons TD, Hoffman RE. Risk for rabies transmission from encounters with bats, Colorado, 1977–1996. Emerging infectious diseases. 1999;5(3):433–7. 10.3201/eid0503.990315 10341181 PMC2640787

[23] Parker EK, Dowda H, Redden SE, Tolson MW, Turner N, Kemick W. Bat rabies in South Carolina, 1970–90. J Wildl Dis. 1999;35(3):557–64. 10.7589/0090-3558-35.3.557 10479091

[24] Wang X, DeMaria A, Smole S, Brown CM, Han L. Bat rabies in Massachusetts, USA, 1985–2009. Emerging infectious diseases. 2010;16(8):1285–8. 10.3201/eid1608.100205 20678326 PMC3298317

[25] Prins B, Loewen K. Bat rabies in British Columbia 1971–1985. Can Vet J. 1988;29(1):41–4. 17422945 PMC1680738

[26] Pybus MJ. Rabies in insectivorous bats of western Canada, 1979 to 1983. J Wildl Dis. 1986;22(3):307–13. 3735577 10.7589/0090-3558-22.3.307

[27] CDC. Protocol for Postmortem Diagnosis of Rabies in Animals by Direct Fluorescent Antibody Testing: Centers for Disease Control and Prevention; 2018 [https://www.cdc.gov/rabies/pdf/rabiesdfaspv2.pdf.

[28] Nagorsen DW, Brigham RM. Bats of British Columbia. Volume 1, The Mammals of British Columbia Royal British Columbia Museum Handbook. Vancouver: University of British Columbia Press; 1993.

[29] Kearse M, Moir R, Wilson A, Stones-Havas S, Cheung M, Sturrock S, et al Geneious Basic: an integrated and extendable desktop software platform for the organization and analysis of sequence data. Bioinformatics. 2012;28(12):1647–9. 10.1093/bioinformatics/bts199 22543367 PMC3371832

[30] Edgar RC. MUSCLE: multiple sequence alignment with high accuracy and high throughput. Nucleic Acids Res. 2004;32(5):1792–7. 10.1093/nar/gkh340 15034147 PMC390337

[31] Kumar S, Stecher G, Tamura K. MEGA7: Molecular Evolutionary Genetics Analysis Version 7.0 for Bigger Datasets. Mol Biol Evol. 2016;33(7):1870–4. 10.1093/molbev/msw054 27004904 PMC8210823

[32] Tamura K, Masatoshi N, Kumar S. Prospects for inferring very large phylogenies by using the neighbor-joining method. Proceedings of the National Academy of Sciences of the United States of America. 2004;101(0027–8424 (Print)). 10.1073/pnas.0404206101 PMC491989 15258291

[33] Felsenstein J. CONFIDENCE LIMITS ON PHYLOGENIES: AN APPROACH USING THE BOOTSTRAP. (1558–5646 (Electronic)). 10.1111/j.1558-5646.1985.tb00420.x 28561359

[34] Klug BJ, Turmelle AS, Ellison JA, Baerwald EF, Barclay RM. Rabies prevalence in migratory tree-bats in Alberta and the influence of roosting ecology and sampling method on reported prevalence of rabies in bats. J Wildl Dis. 2011;47(1):64–77. 10.7589/0090-3558-47.1.64 21269998

[35] Mercaldo ND, Lau Kf Fau—Zhou XH, Zhou XH. Confidence intervals for predictive values with an emphasis to case-control studies. (0277–6715 (Print)). 10.1002/sim.2677 16927452

[36] IDHW. Rabies in Idaho. Idaho Department of Health and Welfare, Division of Public Health; 2017.

[37] OHA. Selected Reportable Communicable Disease Summary 2015. Oregon Health Authority; 2017.

[38] Liesener AL, Smith KE, D R.D., Bender JB, Danila R, Neitzel DF, et al Circumstances of bat encounters and knowledge of rabies among Minnesota residents submitting bats for rabies testing. Vector borne and zoonotic diseases. 2006;6(2):208–15. 10.1089/vbz.2006.6.208 16796518

[39] Picard-Meyer E, Robardet E, Arthur L, Larcher G, Harbusch C, Servat A, et al Bat rabies in France: a 24-year retrospective epidemiological study. PloS one. 2014;9(6):e98622 10.1371/journal.pone.0098622 24892287 PMC4044004

[40] Messenger SL, Smith JS, Rupprecht CE. Emerging epidemiology of bat-associated cryptic cases of rabies in humans in the United States. Clin Infect Dis. 2002;35(6):738–47. 10.1086/342387 12203172

[41] Hayes G, Wiles GJ. Washington State Bat Conservation Plan. Olympia, Washington: Washington Department of Fish and Wildlife; 2013.

[42] Falxa GA. Winter foraging behavior of silver-haired and California myotis bats in western Washington. Northwestern Naturalist. 2007;88:98–100.

[43] George DB, Webb CT, Farnsworth ML, O’Shea TJ, Bowen RA, Smith DL, et al Host and viral ecology determine bat rabies seasonality and maintenance. Proceedings of the National Academy of Sciences of the United States of America. 2011;108(25):10208–13. 10.1073/pnas.1010875108 21646516 PMC3121824

[44] Schowalter DB. Characteristics of bat rabies in Alberta. Can J Comp Med. 1980;44(1):70–6. 7397600 PMC1320036

[45] Turmelle AS, Jackson FR, Green D, McCracken GF, Rupprecht CE. Host immunity to repeated rabies virus infection in big brown bats. J Gen Virol. 2010;91(Pt 9):2360–6. 10.1099/vir.0.020073-0 20519458 PMC3052523

[46] BCI. Species Profiles. Eptesicus fuscus: Bat Conservation International; 2018 [http://www.batcon.org/resources/media-education/species-profiles/detail/1890.

[47] O’Shea TJ, Bowen RA, Stanley TR, Shankar V, Rupprecht CE. Variability in Seroprevalence of Rabies Virus Neutralizing Antibodies and Associated Factors in a Colorado Population of Big Brown Bats (*Eptesicus fuscus*). PloS one. 2014;9(1):e86261 10.1371/journal.pone.0086261 24465996 PMC3899234

[48] Lausen C, Barclay RM. Benefits of living in a building: big brown bats (*Eptesicus fuscus*) in rocks versus buildings. Journal of Mammalogy. 2006;87(2):362–70.

[49] CDC. Human rabies—Montana and Washington, 1997. MMWR Morb Mortal Wkly Rep. 1997(46):770–4. 9272584

[50] CDC. Human rabies—Washington, 1995. MMWR Morb Mortal Wkly Rep. 1995;1(44):625–7. 7643847

[51] Dato VM, Campagnolo ER, Long J, Rupprecht CE. A Systematic Review of Human Bat Rabies Virus Variant Cases: Evaluating Unprotected Physical Contact with Claws and Teeth in Support of Accurate Risk Assessments. PloS one. 2016;11(7):e0159443 10.1371/journal.pone.0159443 27459720 PMC4961291

[52] De Serres G, D F, Côte M, Skowronski DM. Bat rabies in the United States and Canada from 1950 through 2007: human cases with and without bat contact. Clin Infect Dis. 2008;46(9):1329–37. 10.1086/586745 18419432

[53] USDFW. Northern Long-eared Bat Range Map. U.S. Fish & Wildlife Service. 2018 [https://www.fws.gov/Midwest/endangered/mammals/nleb/nlebRangeMap.html.

[54] De Serres G, Skowronski DM, Mimault P, Ouakki M, Maranda-Aubut R, Duval B. Bats in the bedroom, bats in the belfry: reanalysis of the rationale for rabies postexposure prophylaxis. Clin Infect Dis. 2009;48(11):1493–9. 10.1086/598998 19400689

[55] Bemis K, Frias M, Patel MT, Christiansen D. Using an Emergency Department Syndromic Surveillance System to Evaluate Reporting of Potential Rabies Exposures, Illinois, 2013–2015. Public Health Rep. 2017;132:1. 10.1177/0033354917708355 PMC5676512 28692394

[56] Lorch JM, Palmer JM, Lindner DL, Ballmann AE, George KG, Griffin K, et al First Detection of Bat White-Nose Syndrome in Western North America. mSphere. 2016;1(4):e00148–16. 10.1128/mSphere.00148-16 27504499 PMC4973635

[57] Ommundsen P, Lausen C, Matthias L. First Acoustic Records of the Brazilian Free-Tailed Bat (*Tadarida brasiliensis*) In British Columbia. Northwestern Naturalist. 2017;98(2):132–6.

[58] Gigante CM, Dettinger L, Powell JW, Seiders M, Condori REC, Griesser R, et al Multi-site evaluation of the LN34 pan-lyssavirus real-time RT-PCR assay for post-mortem rabies diagnostics. PloS one. 2018;13(5):e0197074 10.1371/journal.pone.0197074 29768505 PMC5955534

