# Supplementary material for: Bat rabies in Washington State: Temporal-spatial trends and risk factors for zoonotic transmission (2000–2017)
Source: PLoS One. 2018 Oct 9;13(10):e0205069. doi: 10.1371/journal.pone.0205069 (PMC6177155; doi:10.1371/journal.pone.0205069)
Supplement: S1 Appendix — (PDF) [file pone.0205069.s001.pdf]

Bat rabies in Washington State: trends and risk factors for infection (2000–2017)
Jesse Bonwitt, Hanna Oltean, Misty Lang, Rochelle M. Kelly, Marcia Goldoft

Methods

Genetic identification of bats

We collected bilateral wing tissue samples using a 4 mm punch biopsy (Integra Miltex, Plainsboro, NJ, USA) and extracted DNA using DNeasy extraction kits (Qiagen, Germantown, MD, USA). We then amplified a 654 base pair fragment of the cytochrome *b* gene by polymerase chain reaction (PCR) using primers F504 and R1181 [1].

PCR was carried out in 25μl reactions, consisting of 8 μl molecular grade water, 12.5 μl Dream Taq Green mastermix (Fisher Scientific, Waltham, MA, USA), 0.5 μM of each primer, and 2 μl of template DNA. The thermocycler profile consisted of 95ºC for 15 min, followed by 35 cycles of 94ºC for 30 sec, 50ºC for 90 sec, and 72ºC for 60 sec, and a final extension at 72ºC for 30 min. We confirmed successful PCR products on a 1% agarose gel stained with SybrGreen (Invitrogen, Carlsbad, CA, USA). Successful PCR products were sent to MC Lab (San Francisco, CA, USA) for PCR clean up and sequencing in both directions on an ABI 3730XL sequencer (Applied Biosystems Inc, Carlsbad, CA, USA). We edited and generated consensus sequences using Geneious version 9.0.4 (Biomatters Inc., Newark, NJ, USA) and aligned sequences using the MUSCLE alignment [2] in MEGA 7 [3]. We compared sequences to reference sequences using the NCBI Basic Local Alignment Search Tool to identify species.

Amplification of cytochrome *b* does not allow differentiation between California myotis (*Myotis californicus*) and small-footed myotis (*Myotis ciliolabrum*) as well as western long-eared myotis (*Myotis evotis*), Keen's myotis (*Myotis keenii*), and fringed myotis (*Myotis thysanodes*).

References

1. Dixon MD. Population genetic structure and natal philopatry in the widespread North American bat Myotis lucifugus. Journal of Mammalogy. 2011;92(6):1343-51.
2. Edgar RC. MUSCLE: multiple sequence alignment with high accuracy and high throughput. Nucleic Acids Res. 2004;32(5):1792-7.
3. Kumar S, Stecher G, Tamura K. MEGA7: Molecular Evolutionary Genetics Analysis Version 7.0 for Bigger Datasets. Mol Biol Evol. 2016;33(7):1870-4.
4. Schowalter DB. Characteristics of bat rabies in Alberta. Can J Comp Med. 1980;44(1):70-6.
5. Beauregard M. Bat rabies in Canada 1963-1967. Can J Comp Med. 1969;33(3):220-6.
6. Prins B, Loewen K. Bat rabies in British Columbia 1971-1985. Can Vet J. 1988;29(1):41-4.
