# Supplementary material for: Bat rabies in Washington State: Temporal-spatial trends and risk factors for zoonotic transmission (2000–2017)
Source: PLoS One. 2018 Oct 9;13(10):e0205069. doi: 10.1371/journal.pone.0205069 (PMC6177155; doi:10.1371/journal.pone.0205069)
Supplement: S1 Table — Data from Klug et al (2011)± and Hayes et al (2013)§. (PDF) [file pone.0205069.s002.pdf]

Table S1. Classification of bats according to synanthropic status. Data from Klug et al (2011)[±] and Hayes et al (2013)[§].

Species	Synanthropic
Big brown bat (*Eptesicus fuscus)*	Yes[±]
Silver-haired bat (*Lasionycteris noctivagans*)	No[±]
California myotis (*Myotis californicus*)	Yes[±]
Western long-eared myotis (*Myotis evotis*)	No[±]
Yuma myotis (*Myotis yumanensis*)	Yes[±]
Little brown bat (*Myotis lucifugus*)	Yes[±]
Hoary bat (*Lasiurus cinereus*)	No[±]
Northern long-eared myotis (*Myotis septentrionalis*)[¶]	No[±]
Fringed myotis (*Myotis thysanodes*)	Yes[±]
Townsend's big-eared bat (*Corynorhinus townsendii*)	No[±]
Spotted bat (*Euderma maculatum*)	No[±]
Southern red bat (*Lasiurus blossevillii*)[¶]	Unk
Small-footed myotis (*Myotis ciliolabrum*)	Yes[§]
Keen's myotis (*Myotis keenii*)	No[±]
Long-legged myotis (*Myotis volans*)	Yes[±]
Big free-tailed bat (*Nyctinomops macrotis*)[¶]	No[±]
Pallid bat (*Antrozous pallidus*)	No[±]
Canyon bat (*Parastrellus hesperus*)	No[±]
