# Supplementary material for: Bat rabies in Washington State: Temporal-spatial trends and risk factors for zoonotic transmission (2000–2017)
Source: PLoS One. 2018 Oct 9;13(10):e0205069. doi: 10.1371/journal.pone.0205069 (PMC6177155; doi:10.1371/journal.pone.0205069)
Supplement: S2 Table — (PDF) [file pone.0205069.s003.pdf]

Table S2. Definitions for circumstances of encounters between bats and humans, cats, and dogs.

Variable	Mutually exclusive with:	Definition
**Place of Bat Encounter**		
Found in house	Found outdoors<br>Found in other structure	Bat was found inside a house. This excludes instances where a pet animal brings a bat into the house from outside.
Found in other structure	Found outdoors<br>Found in house	Bat was found in an indoor structure that is not a house. E.g. cabin, church, warehouse, building under construction, barn, garage, school, hospital, store, or car.
Found outdoors	Found in house<br>Found in other structure	Bat was found outside of a house or other human built structure.
**Bat-Pet Interaction**		
Caught by cat	Caught by dog<br>Unknown<br>Other	Bat was caught by cat. If known, indicate if the cat caught the bat outdoors or indoors.
Caught by dog	Caught by cat<br>Unknown<br>Other	Bat was caught by dog. If known, indicate if the dog caught the bat outdoors or indoors.
**Vital Status**		
Found dead	Unknown<br>Other	Bat is found dead. Excludes events where a human kills a bat or a pet is seen killing a bat.
Inflight collision	Found dead<br>All except (others, unknown)	Bat collides with human during flight but does not remain attached, except if tangled in hair.
**Bat Signs and Behaviors**		
Abnormal behavior	Found dead<br>Unknown<br>Other	Bat initiates contact with human. Includes flying into human and remaining attached. Excludes inflight collision.
Abnormal hiding	Found dead<br>Unknown<br>Other	Bat is found in a hiding place at ground level. E.g. shoes, bin, clothes basket, bra, glove, or newspaper.
Injured/sick	Found dead<br>Unknown<br>Other	Bat specifically reported as injured/sick; bat cared for at a wildlife rehabilitation; human can pick up bat without having to catch it in flight. The bat does not initiate contact.
Bat bite	Found dead<br>Unknown<br>Other	Human bitten by a bat. Excludes instances where human bears bite marks but did not witness the bite. If known, specify if bite was provoked or unprovoked.
Drowned	Unknown<br>Other	Bat is found (alive or dead) in a body of water. E.g. pools, barrels, water bowls, or sinks.
Other	All except unknown	
Unknown	All except others	Unknown bat behavior.
