# Supplementary material for: Bat rabies in Washington State: Temporal-spatial trends and risk factors for zoonotic transmission (2000–2017)
Source: PLoS One. 2018 Oct 9;13(10):e0205069. doi: 10.1371/journal.pone.0205069 (PMC6177155; doi:10.1371/journal.pone.0205069)
Supplement: S3 Table — A space-time cluster was defined as ≥4 RABV positive bats originating from proximal counties during contiguous months. (PDF) [file pone.0205069.s004.pdf]

Table S3. Bats with positive RABV test results by cluster, Washington State— 2006–2017. A space-time cluster was defined as ≥4 RABV positive bats originating from proximal counties during contiguous months.

Year	Month	Date	Species	Cluster Size	County
2008	June	6/4/2008	*E. fuscus*	4	Pierce
	July	7/9/2008			King
	July	7/18/2008			Thurston
	August	8/5/2008			Snohomish
2009	July	7/14/2009	*E. fuscus*	4	King
	August	8/4/2009			Pierce
	September	9/3/2009			Thurston
	October	10/7/2009			Kittitas
2014	August	8/15/2014	*M. evotis*	4	Clallam
	August	8/7/2014			Skagit
	September	9/18/2014			Kitsap
	October	10/3/2014			Kitsap
2015	June	6/22/2015	*E. fuscus*	5	Thurston
	July	7/27/2015			King
	August	8/5/2015			Mason
	September	9/21/2015			King
	September	9/25/2015			Snohomish
2016	July	7/22/2016	*E. fuscus*	4	Mason
	August	8/22/2016			Lewis
	September	9/20/2016			Pierce
	September	9/30/2016			Thurston
2017	May	5/16/2017	*E. fuscus*	11	Snohomish
	June	6/6/2017			Pierce
	July	7/26/2017			Snohomish
	July	7/21/2017			King
	August	8/31/2017			King
	August	8/30/2017			King
	August	8/24/2017			King
	August	8/18/2017			King
	August	8/29/2017			Lewis
	August	8/25/2017			Pierce
	September	9/6/2017			King
