# Supplementary material for: Bat rabies in Washington State: Temporal-spatial trends and risk factors for zoonotic transmission (2000–2017)
Source: PLoS One. 2018 Oct 9;13(10):e0205069. doi: 10.1371/journal.pone.0205069 (PMC6177155; doi:10.1371/journal.pone.0205069)
Supplement: S4 Table — (PDF) [file pone.0205069.s005.pdf]

Table S4. RABV positivity (by fluorescent antibody testing) among passively sampled big brown bats in Southern Canada, 1963–1985.

Geographic area	Date	No. bats tested	No. bats positive	% bats positive	Reference
Alberta	1973–1978	434	34	7.8	[4]
British Columbia	1963–1967	32	7	22.0	[5]
British Columbia	1977–1985	197	33	16.8	[6]
Ontario	1963–1967	220	25	11.4	[5]
