# Supplementary material for: Bat rabies in Washington State: Temporal-spatial trends and risk factors for zoonotic transmission (2000–2017)
Source: PLoS One. 2018 Oct 9;13(10):e0205069. doi: 10.1371/journal.pone.0205069 (PMC6177155; doi:10.1371/journal.pone.0205069)
Supplement: S1 Fig — (PDF) [file pone.0205069.s006.pdf]

Bat rabies in Washington State: trends and risk factors for infection (2000–2017)
Jesse Bonwitt, Hanna Oltean, Misty Lang, Rochelle M. Kelly, Marcia Goldoft

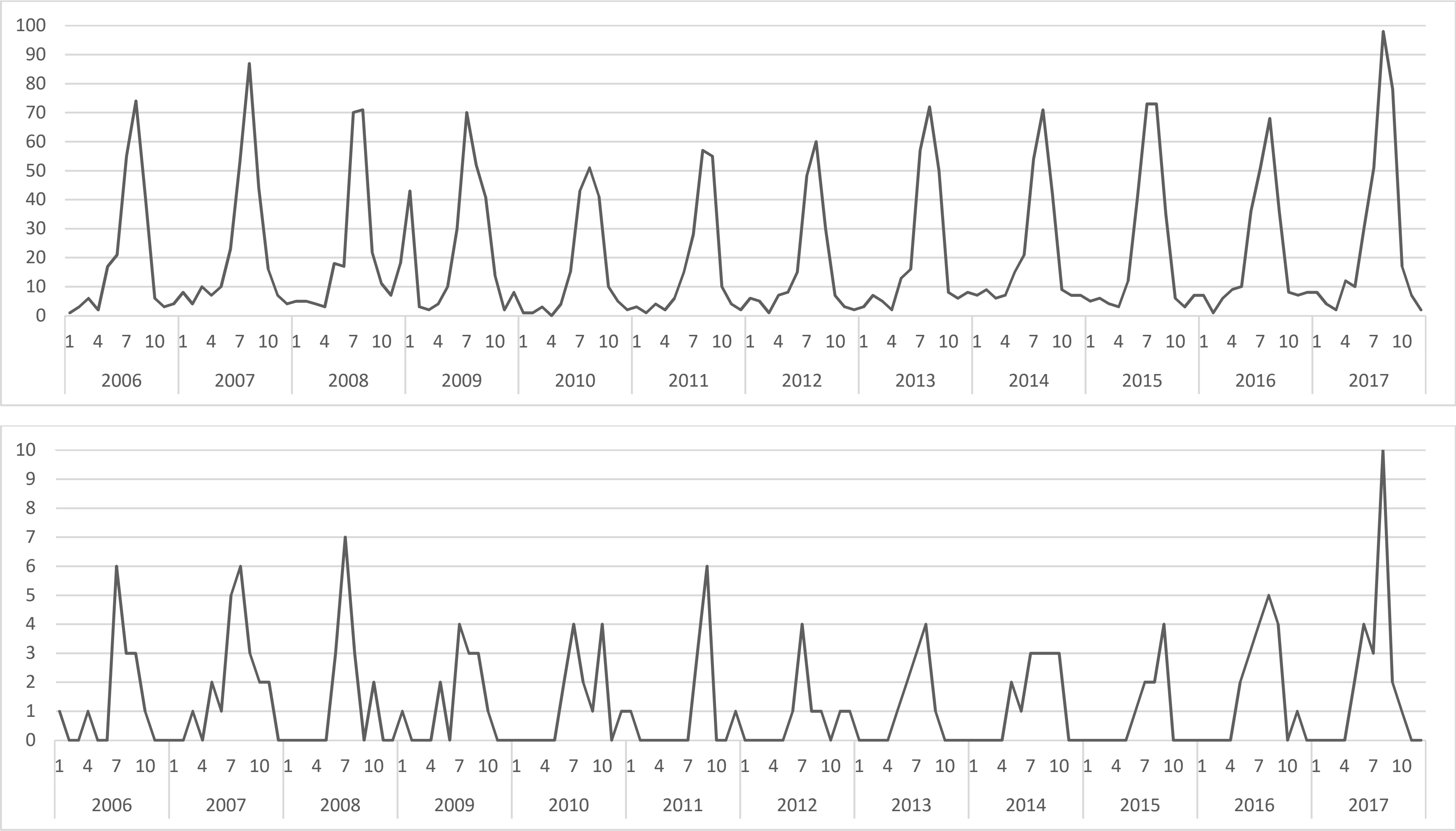

Figure S1. Numbers of identified bats tested with definitive RABV test results (upper figure) and number of identified bats positive for RABV (lower figure) by month and year, Washington State — 2006–2017.
