# Supplementary material for: Bat rabies in Washington State: Temporal-spatial trends and risk factors for zoonotic transmission (2000–2017)
Source: PLoS One. 2018 Oct 9;13(10):e0205069. doi: 10.1371/journal.pone.0205069 (PMC6177155; doi:10.1371/journal.pone.0205069)
Supplement: S2 Fig — (PDF) [file pone.0205069.s007.pdf]

Bat rabies in Washington State: trends and risk factors for infection (2000–2017)
Jesse Bonwitt, Hanna Oltean, Misty Lang, Rochelle M. Kelly, Marcia Goldoft

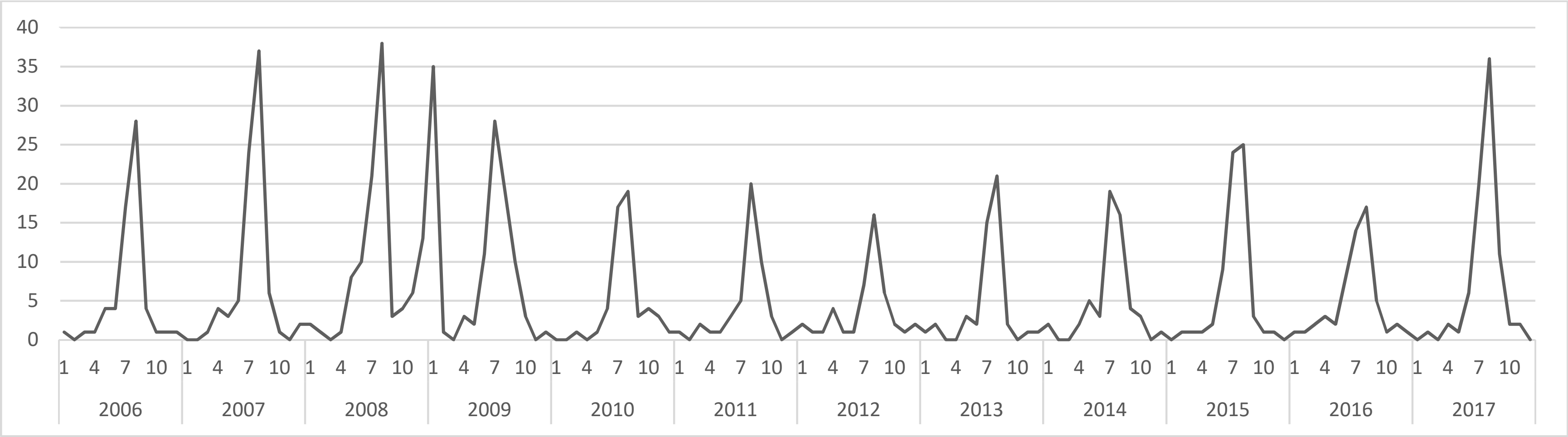

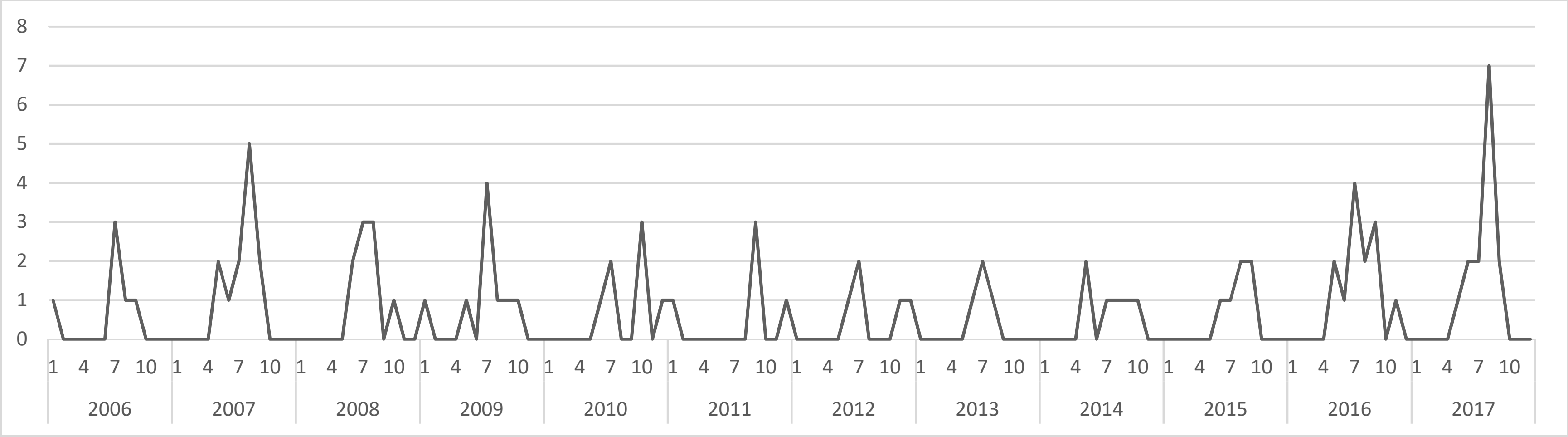

Figure S2A. Numbers of big brown bats (*Eptesicus fuscus*) tested with definitive RABV test results (upper figure) and number of bats positive for RABV (lower figure) by month and year, Washington State — 2006–2017.

Bat rabies in Washington State: trends and risk factors for infection (2000–2017)
Jesse Bonwitt, Hanna Oltean, Misty Lang, Rochelle M. Kelly, Marcia Goldoft

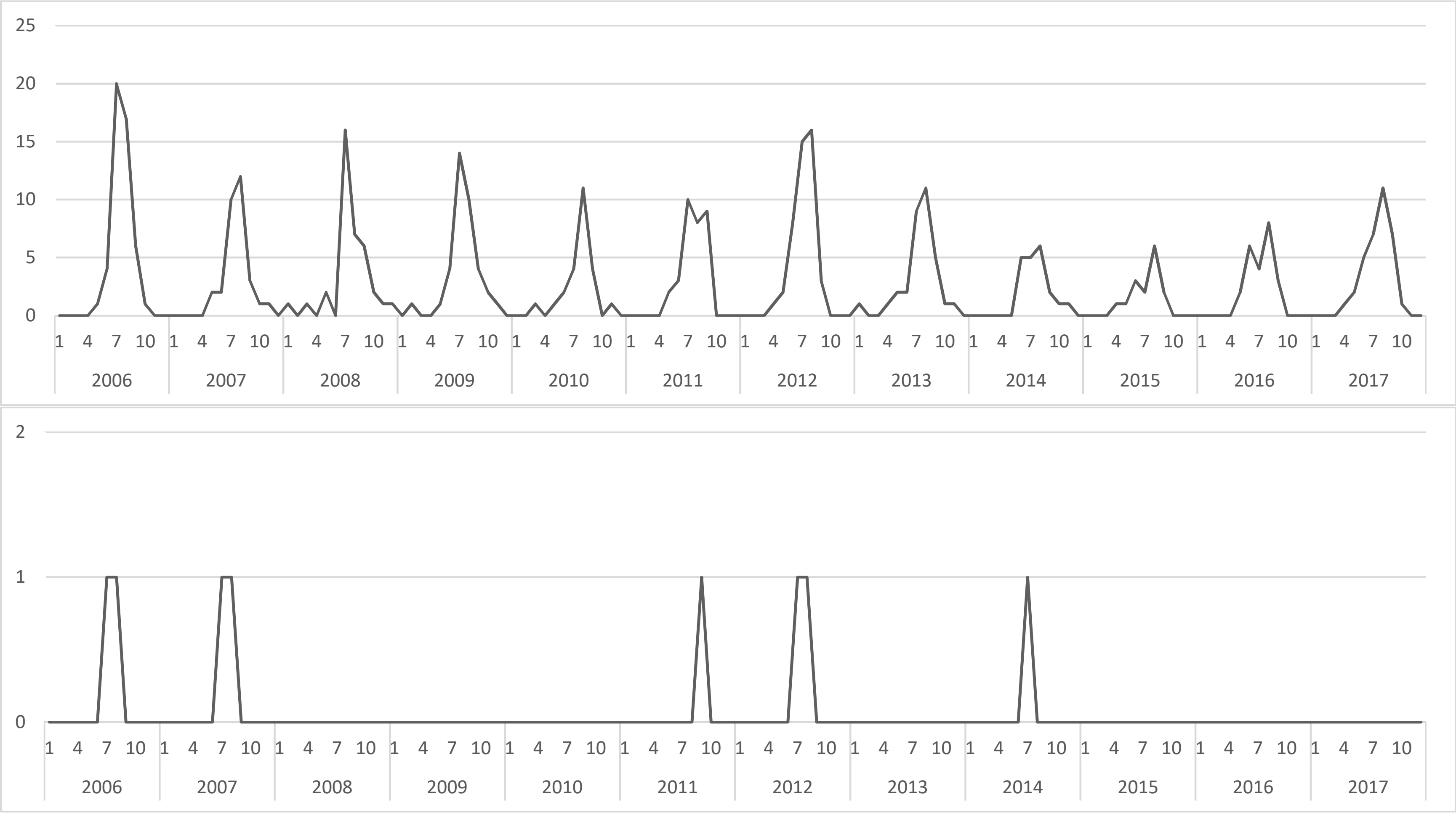

Figure S2B. Numbers of little brown bats (*Myotis lucifugus*) tested with definitive RABV test results (upper figure) and number of bats positive for RABV (lower figure) by month and year, Washington State — 2006–2017.

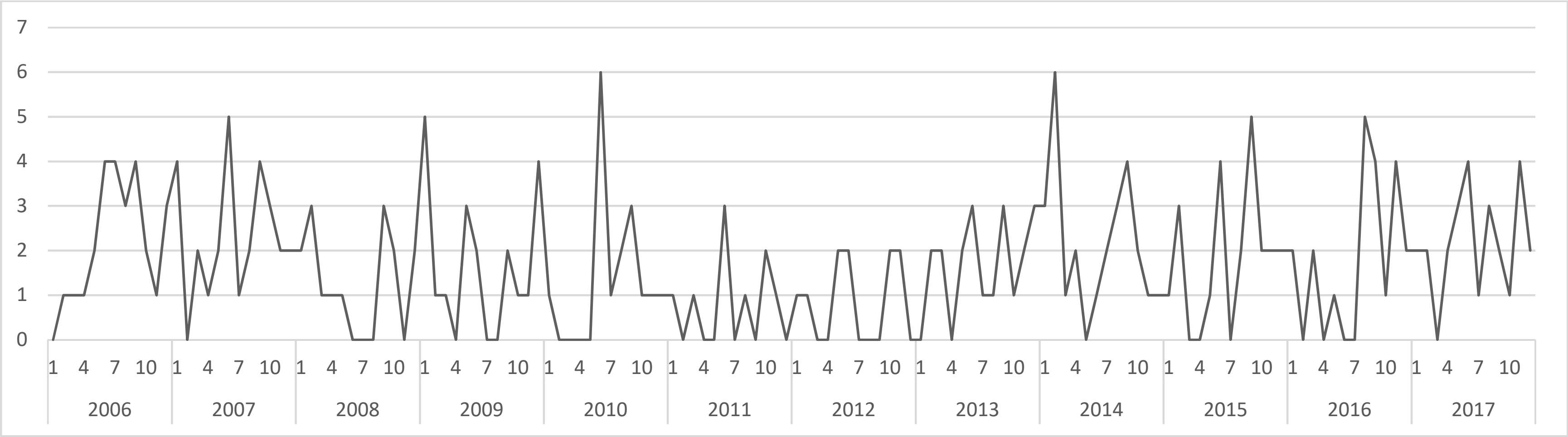

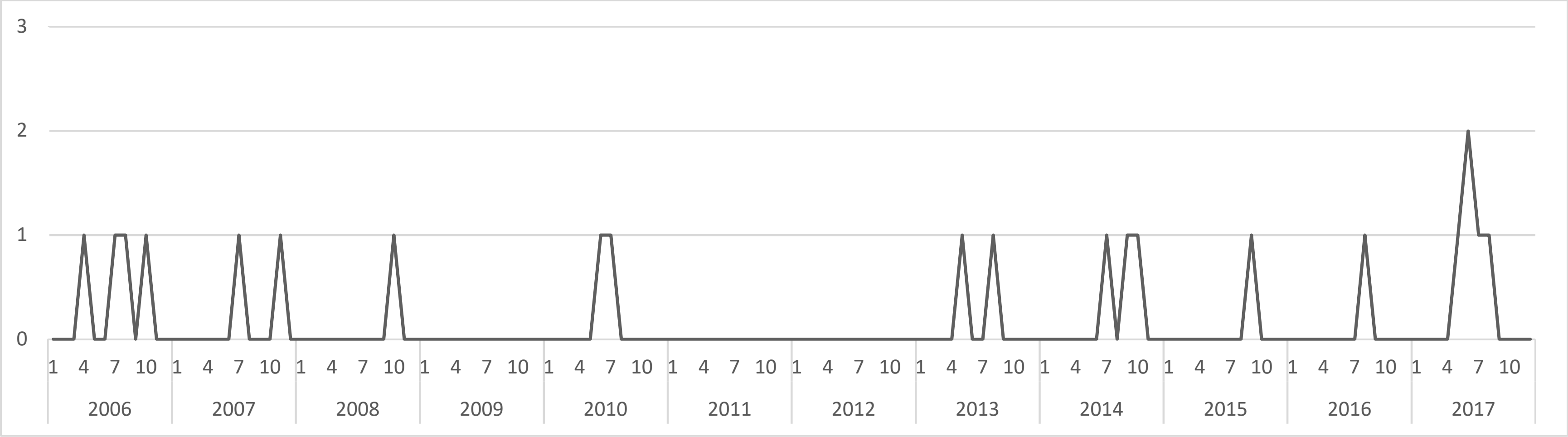

Figure S2C. Numbers of silver-haired bats (*Lasionycteris noctivagans*) tested with definitive RABV test results (upper figure) and number of bats positive for RABV (lower figure) by month and year, Washington State — 2006–2017.

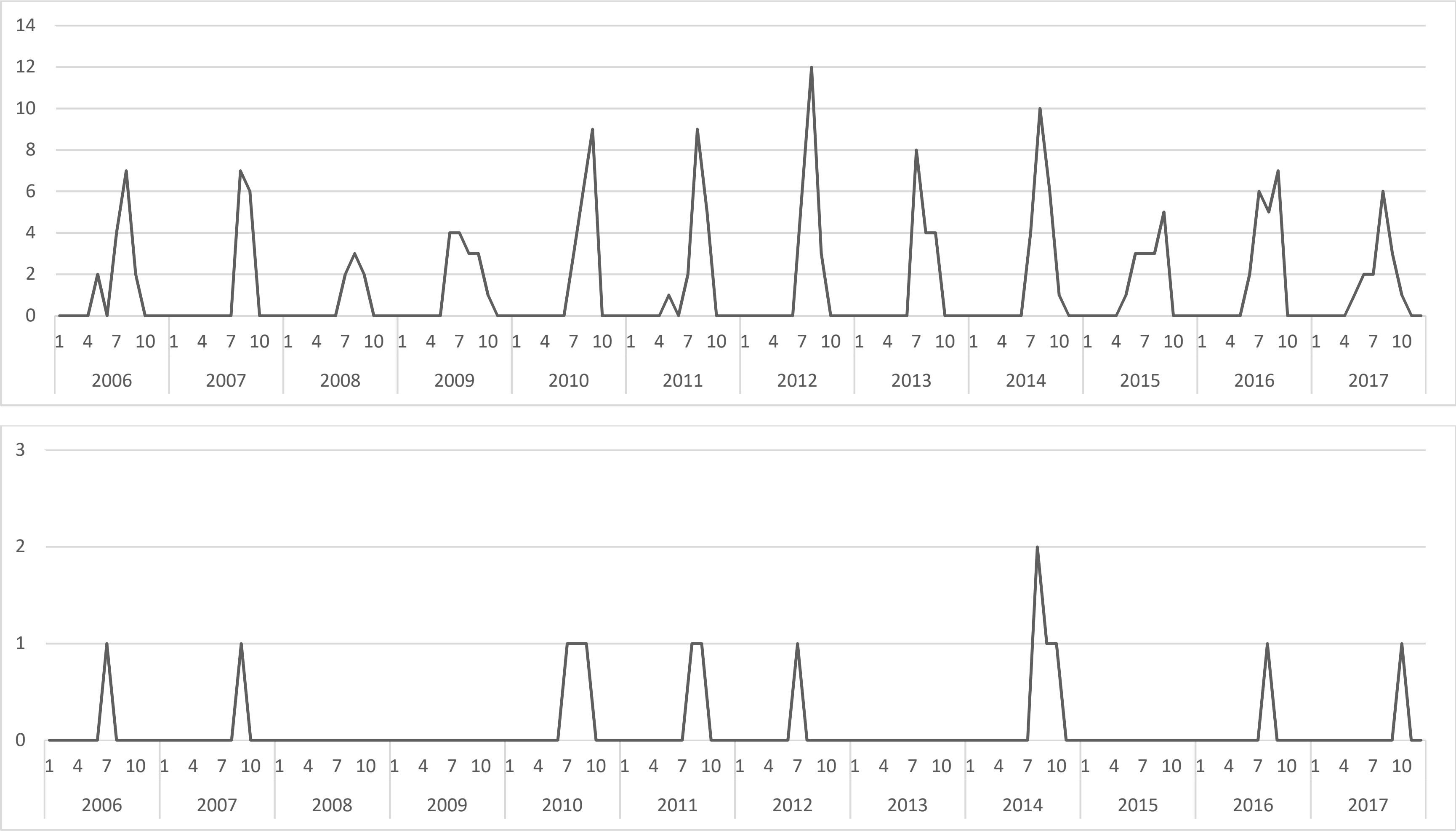

Figure S2D. Numbers of western long-eared myotis (*Myotis evotis*) tested with definitive RABV test results (upper figure) and number of bats positive for RABV (lower figure) by month and year, Washington State — 2006–2017.

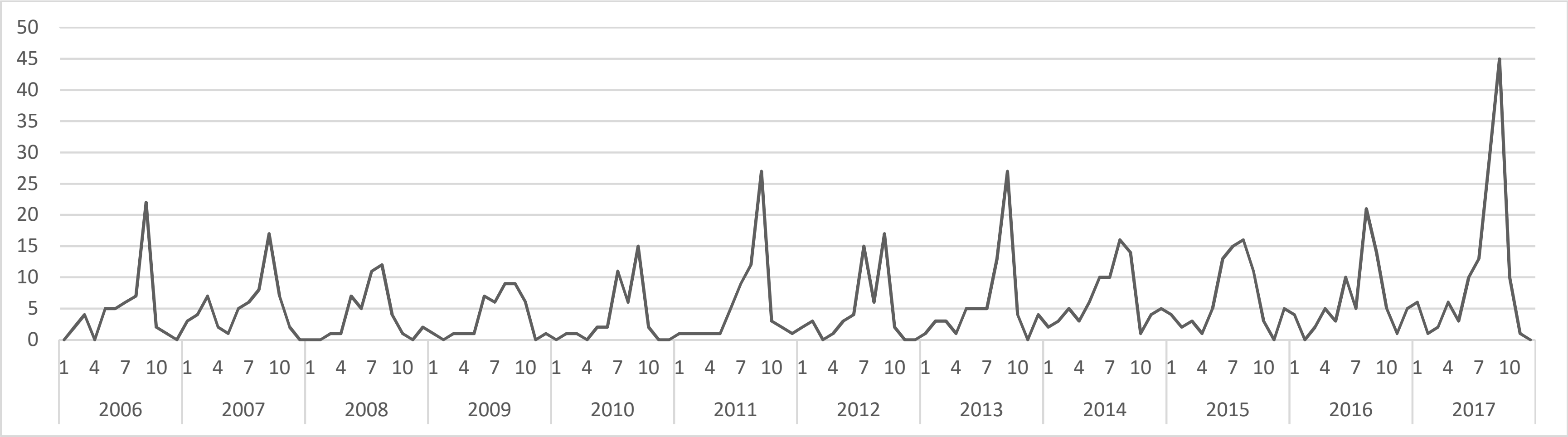

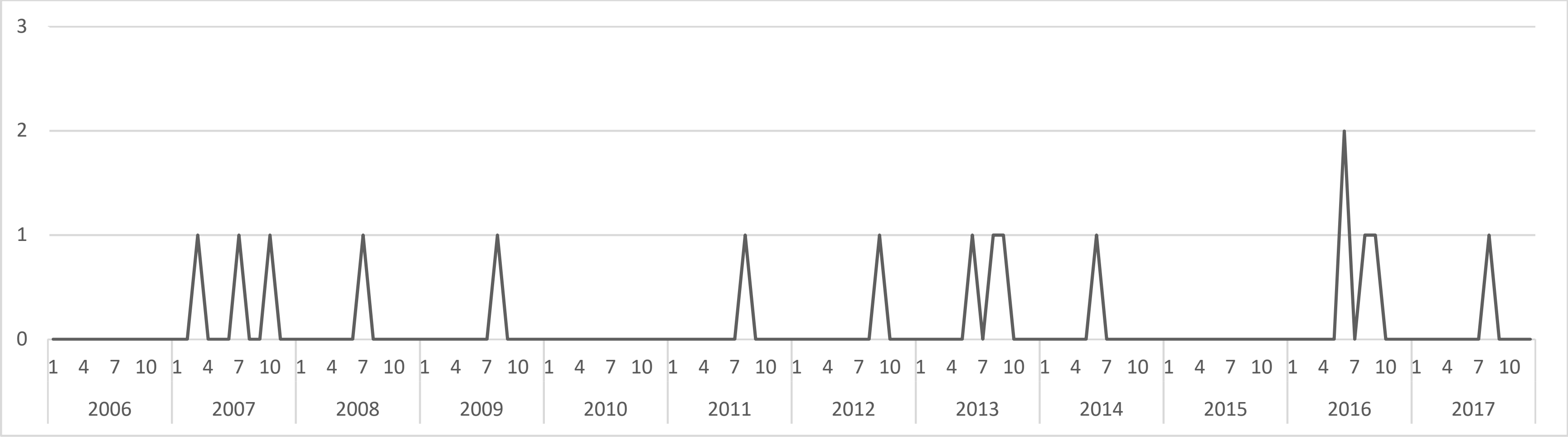

Figure S2E. Numbers of California myotis (*Myotis californicus*) tested with definitive RABV test results (upper figure) and number of bats positive for RABV (lower figure) by month and year, Washington State — 2006–2017.

Bat rabies in Washington State: trends and risk factors for infection (2000–2017)
Jesse Bonwitt, Hanna Oltean, Misty Lang, Rochelle M. Kelly, Marcia Goldoft

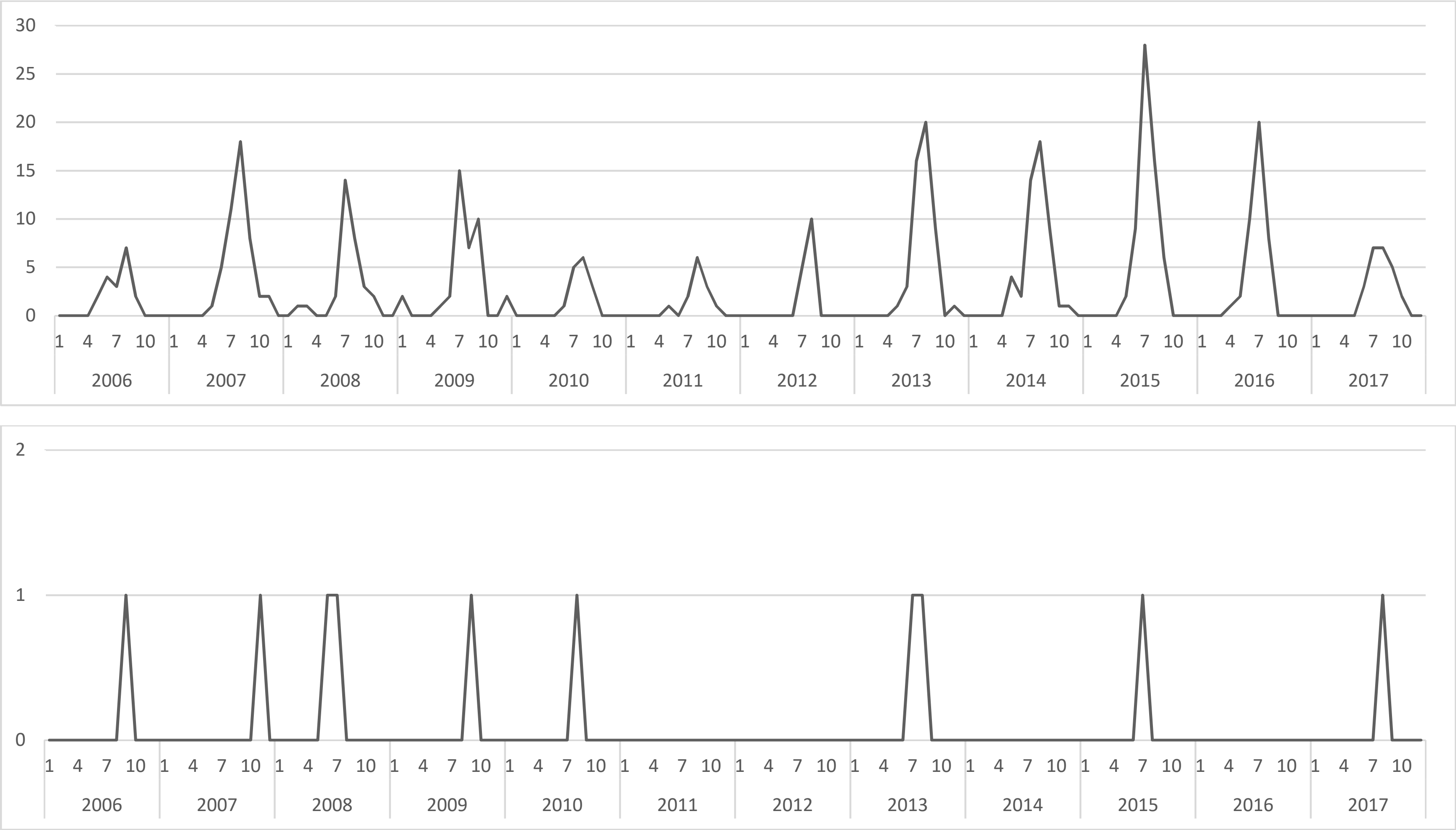

Figure S2F. Numbers of Yuma myotis (*Myotis yumanensis*) tested with definitive RABV test results (upper figure) and number of bats positive for RABV (lower figure) by month and year, Washington State — 2006–2017.
