# Supplementary material for: Bat rabies in Washington State: Temporal-spatial trends and risk factors for zoonotic transmission (2000–2017)
Source: PLoS One. 2018 Oct 9;13(10):e0205069. doi: 10.1371/journal.pone.0205069 (PMC6177155; doi:10.1371/journal.pone.0205069)
Supplement: S3 Fig — Shapes denote month of submission; triangle: May; diamond: June; square: July, circle: August. (PDF) [file pone.0205069.s008.pdf]

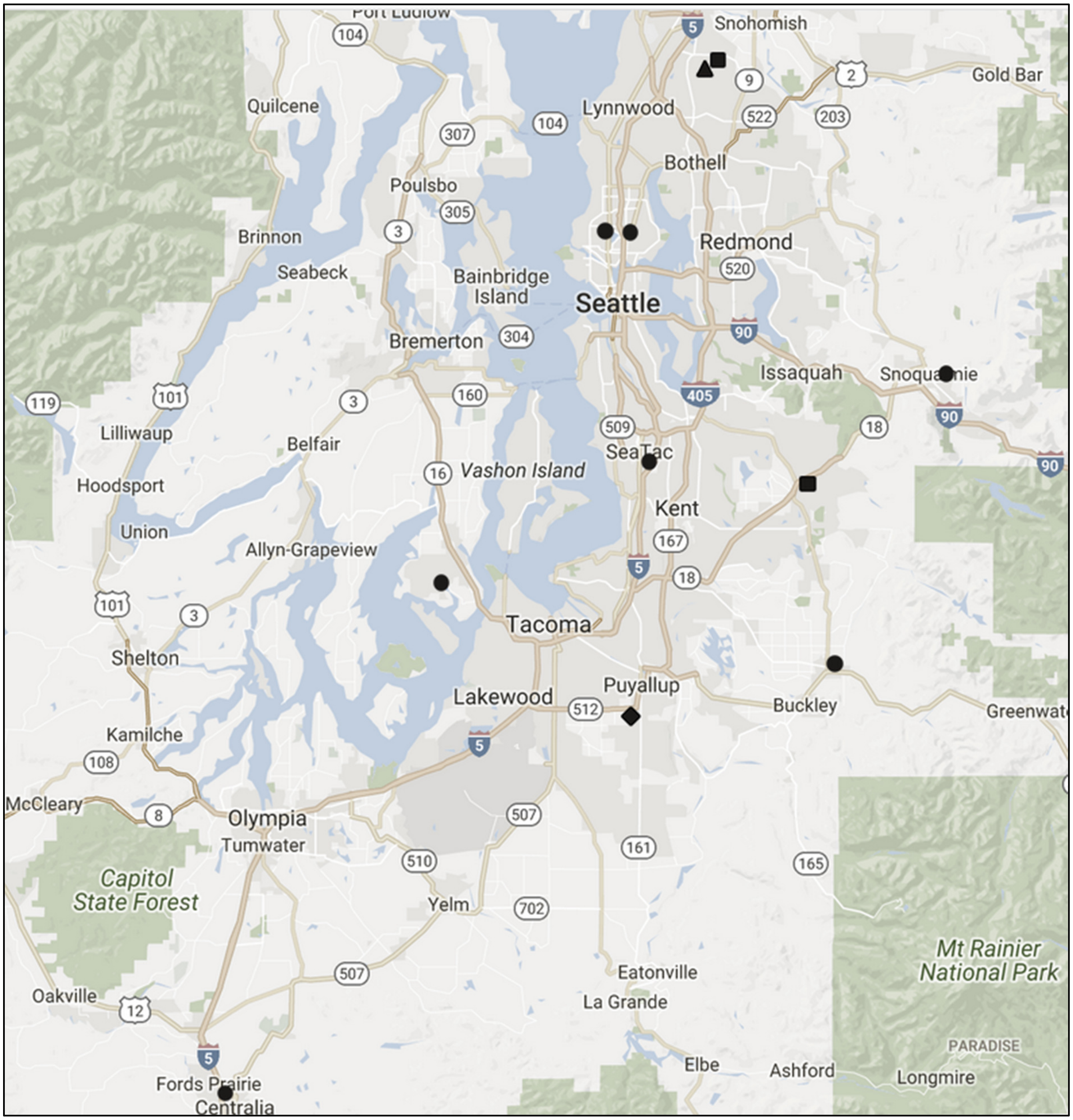

Figure S3. Cluster of RABV positive big brown bats (*Eptesicus fuscus*) in 2017 (N=11), Western Washington. Shapes denote month of submission; triangle: May; diamond: June; square: July, circle: August.
