# Supplementary material for: Bat rabies in Washington State: Temporal-spatial trends and risk factors for zoonotic transmission (2000–2017)
Source: PLoS One. 2018 Oct 9;13(10):e0205069. doi: 10.1371/journal.pone.0205069 (PMC6177155; doi:10.1371/journal.pone.0205069)
Supplement: S4 Fig — A neighbor-joining tree generated for 97 CytB sequences of 629bp in length. The 73 Myotis spp. sequences generated in this study were morphologically identified by the Department of Health and are indicated by their sample IDs, whereas the 24 reference sequences are indicated by their GenBank accession numbers and species names. Species assigned to each major clade are indicated, and bootstrap values greater than 70% are indicated. Individuals that clustered in clades outside of their morphological assignment are indicated by (*). (PDF) [file pone.0205069.s009.pdf]

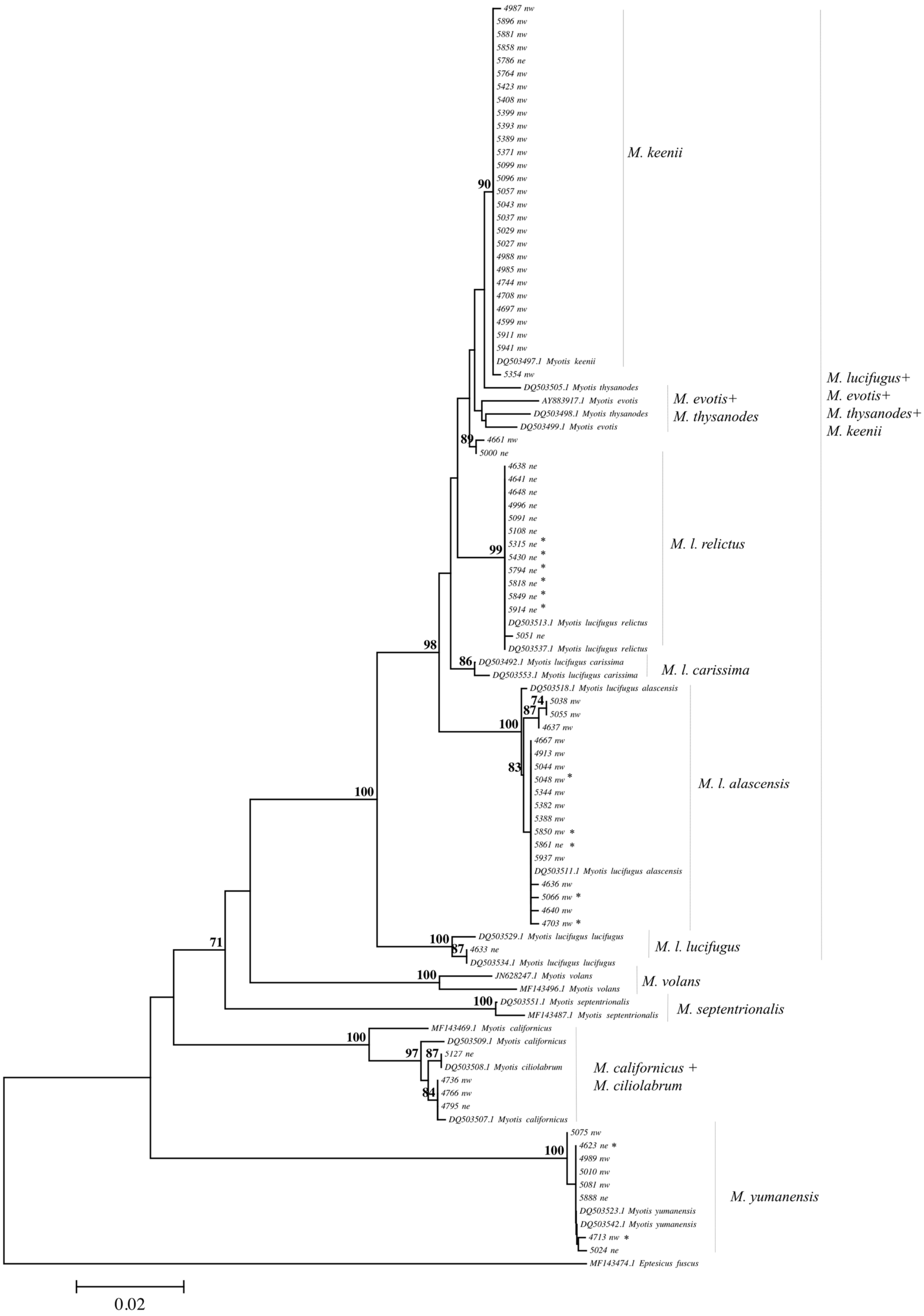
4987 nw
5896 nw
5881 nw
5858 nw
5786 ne
5764 nw
5423 nw
5408 nw
5399 nw
5393 nw
5389 nw
5371 nw
5099 nw
5096 nw
5057 nw
5043 nw
5037 nw
5029 nw
5027 nw
4988 nw
4985 nw
4744 nw
4708 nw
4697 nw
4599 nw
5911 nw
5941 nw
DQ503497.1 Myotis keenii
5354 nw
90
M. keenii
DQ503505.1 Myotis thysanodes
AY883917.1 Myotis evotis
DQ503498.1 Myotis thysanodes
DQ503499.1 Myotis evotis
M. evotis+
M. thysanodes
89
4661 nw
5000 ne
4638 ne
4641 ne
4648 ne
4996 ne
5091 ne
5108 ne
5315 ne *
5430 ne *
5794 ne *
5818 ne *
5849 ne *
5914 ne *
DQ503513.1 Myotis lucifugus relictus
5051 ne
DQ503537.1 Myotis lucifugus relictus
99
M. l. relictus
98
86
DQ503492.1 Myotis lucifugus carissima
DQ503553.1 Myotis lucifugus carissima
M. l. carissima
DQ503518.1 Myotis lucifugus alascensis
74
87
100
5038 nw
5055 nw
4637 nw
4667 nw
4913 nw
5044 nw
5048 nw *
5344 nw
5382 nw
5388 nw
5850 nw *
5861 ne *
5937 nw
DQ503511.1 Myotis lucifugus alascensis
4636 nw
5066 nw *
4640 nw
4703 nw *
83
M. l. alascensis
M. lucifugus+
M. evotis+
M. thysanodes+
M. keenii
100
100
87
DQ503529.1 Myotis lucifugus lucifugus
4633 ne
DQ503534.1 Myotis lucifugus lucifugus
M. l. lucifugus
71
100
JN628247.1 Myotis volans
MF143496.1 Myotis volans
M. volans
100
DQ503551.1 Myotis septentrionalis
MF143487.1 Myotis septentrionalis
M. septentrionalis
100
MF143469.1 Myotis californicus
97
87
DQ503509.1 Myotis californicus
5127 ne
DQ503508.1 Myotis ciliolabrum
4736 nw
4766 nw
4795 ne
84
DQ503507.1 Myotis californicus
M. californicus +
M. ciliolabrum
100
5075 nw
4623 ne *
4989 nw
5010 nw
5081 nw
5888 ne
DQ503523.1 Myotis yumanensis
DQ503542.1 Myotis yumanensis
4713 nw *
5024 ne
M. yumanensis
MF143474.1 Eptesicus fuscus
0.02

Figure S4. *Myotis* spp. (n=73) neighbor-joining phylogenetic tree. A neighbor-joining tree generated for 97 CytB sequences of 629bp in length. The 73 *Myotis* spp. sequences generated in this study were morphologically identified by the Department of Health and are indicated by their sample IDs, whereas the 24 reference sequences are indicated by their GenBank accession numbers and species names. Species assigned to each major clade are indicated, and bootstrap values greater than 70% are indicated. Individuals that clustered in clades outside of their morphological assignment are indicated by (*).
